# Can Double Fortification of Salt with Iron and Iodine Reduce Anemia, Iron Deficiency Anemia, Iron Deficiency, Iodine Deficiency, and Functional Outcomes? Evidence of Efficacy, Effectiveness, and Safety

**DOI:** 10.1093/jn/nxaa192

**Published:** 2021-02-15

**Authors:** Leila M Larson, Shruthi Cyriac, Eric W Djimeu, Mduduzi N N Mbuya, Lynnette M Neufeld

**Affiliations:** University of South Carolina, Department of Health Promotion, Education, and Behavior, Arnold School of Public Health, Columbia, SC, USA; Emory University, Doctoral Program in Nutrition and Health Sciences, Laney Graduate School, Atlanta, GA, USA; Global Alliance for Improved Nutrition, Geneva, Switzerland; Global Alliance for Improved Nutrition, Geneva, Switzerland; Global Alliance for Improved Nutrition, Geneva, Switzerland

**Keywords:** double fortified salt, hemoglobin, iron, anemia, cognition, work productivity, review

## Abstract

**Background:**

Anemia, iron deficiency, and iodine deficiency are problems of important public health concern in many parts of the world, with consequences for the health, development, and work capacity of populations. Several countries are beginning to implement double fortified salt (DFS) programs to simultaneously address iodine and iron deficiencies.

**Objective:**

Our objective was to summarize the evidence for efficacy and effectiveness of DFS on the full range of status and functional outcomes and across different implementation and evaluation designs essential to successful interventions.

**Methods:**

We conducted a systematic review and meta-analysis of published and gray literature examining the effects of DFS on nutritional status, cognition, work productivity, development, and morbidity of all population groups. We searched for articles in Medline, Embase, CINAHL, Cochrane Central Register, and ProQuest for randomized trials, quasi-randomized trials, and program effectiveness evaluations.

**Results:**

A total of 22 studies (N individuals = 52,758) were included. Efficacy studies indicated a significant overall positive effect on hemoglobin concentration [standardized mean difference (95% CI): 0.33 (0.18, 0.48)], ferritin [0.42 (0.08, 0.76)], anemia [risk ratio (95% CI): 0.80 (0.70, 0.92)], and iron deficiency anemia [0.36 (0.24, 0.55)]. Effects on urinary iodine concentration were not significantly different between DFS and iodized salt. The impact on functional outcomes was mixed. Only 2 effectiveness studies were identified. They reported programmatic challenges including low coverage, suboptimal DFS quality, and storage constraints.

**Conclusions:**

Given the biological benefits of DFS across several populations in efficacy research, additional evaluations of robust DFS programs delivered at scale, which consider effective implementation and measure appropriate biomarkers, are needed.

## Introduction

Approximately 43% of children aged under 5 y and 29% of nonpregnant women of reproductive age worldwide are anemic (1). The prevalence varies substantially across countries and is particularly high in India (2). The etiology of anemia is complex and the relative contribution of infectious, genetic, and nutritional causes are not well understood in all contexts; on average across countries, it is estimated that a significant portion of anemia (∼25%) is iron deficiency anemia (IDA) (3). Iron deficiency (ID) in the absence of anemia is also highly prevalent in some contexts (e.g. the USA) (4–6). Supplementation with iron syrup or tablets and home fortification with iron-containing multiple micronutrient powders (MNPs), have demonstrated improvements in iron status and hemoglobin concentrations, particularly in populations with a high prevalence of anemia (7–9). However, supplementation programs are usually best suited to increase consumption in target groups over short periods of time (e.g. pregnancy) and do not address the fundamental issue of consistent low iron intake. Furthermore, consumption of the higher levels of iron often used in supplements may pose risks in some populations (10–12).

Fortification of commonly consumed staple foods to increase the consistent intake of iron could improve status and minimize risks associated with higher iron intake. Several fortification vehicles have been tested, such as wheat and maize flour, and hold promise in populations that consume these regularly (13). In contexts where these are not staples however, double fortified salt (DFS), salt fortified with iron and iodine, could be an appropriate alternative fortification vehicle. Several studies have shown efficacy of DFS on iron status indicators and programs have now been implemented in some contexts. A meta-analysis by Ramirez-Luzuriaga et al. (14) examined DFS efficacy and randomized effectiveness studies. However, 3 developments and considerations necessitate the current study. First, there are now several additional studies that need to be considered; second, quasi-experimental program evaluations are worth considering in assessing effects; and third, effects on iron status, iodine, cognition, work productivity, and potential adverse effects have not been comprehensively reviewed.

There are several programmatic factors that may influence potential for impact including characteristics of the product itself and several implementation considerations. For instance, the stability of iron and acceptability of DFS can vary substantially by formulation (15, 16). The concentration of iron in DFS may influence whether sufficient iron is delivered to overcome deficiency. Finally, supply chain and distribution considerations may limit or facilitate the population's ability and willingness to regularly acquire sufficient DFS to meet their salt use needs. Our research therefore had 2 objectives. First, to review the literature on effects of DFS on hemoglobin concentration, anemia, and IDA prevalence, iron status, iodine, child development, cognition, work productivity, and morbidity outcomes, including recent published and unpublished studies not previously reviewed. Our second objective was to critically assess the internal and external validity of the studies included in the review to identify study/evaluation designs and elements of implementation essential to successful DFS programs.

## Methods

### Inclusion and exclusion criteria

The following predefined criteria were used to select studies for inclusion in this review. Study designs included randomized trials, quasi-randomized trials (i.e. research designs where the intervention group and the comparison group are not generated through a random assignment), program effectiveness evaluations using varying designs including cluster randomized, pre-post designs, with analytical robust methods (difference in difference, instrumental variables estimation, propensity score matching, and regression discontinuity). Only studies that included an intervention group given DFS were included; this excluded all studies using multiple micronutrient-fortified salt, for example. Outcomes of interest included anemia, IDA, ID, hemoglobin, iodine status, iron status, child development, cognition, work productivity, and morbidity. Control groups may have received no intervention or iodized salt. No language or date restrictions were applied.

### Search strategy

In November 2019, we searched MEDLINE (Ovid), Embase (Ovid), CINAHL (EBSCO), Cochrane Central Register of Controlled Trials, and ProQuest (theses and dissertations). The following search terms were used in our database search strategy: double fortified salt, dual fortified salt, dual salt, double salt, iodine and iron, anemia, iron, ferritin, hemoglobin, iron deficiency anemia, haemoglobin, anaemia, iron deficiency anaemia, Bayley, Peabody Picture Vocabulary Test, language, cognitive, cognition, socio-emotional, mental development, psychomotor, motor, sensorimotor, intelligence, IQ, executive function, memory, attention, learning, information processing, literacy, reading, math, school readiness, emotion, productivity, trial, intervention, RCT, program, effectiveness, randomized, experimental, difference in difference, double difference, instrumental variables estimation, propensity score matching, regression discontinuity design. We searched through references of included studies to identify any studies we may have missed from the database search. Furthermore, key organizations working in nutrition were contacted with requests for relevant reports or gray literature.

This review was registered at Prospero, the International prospective register of systematic reviews, as PROSPERO 2019 CRD42019129302.

### Data extraction

Two reviewers independently screened titles and abstracts of articles identified through the search strategy. Following a review of abstracts, full texts were read to confirm inclusion and exclusion criteria. The same 2 reviewers extracted relevant information from all included articles. The following information was extracted from all studies: *1*) country, *2*) population, *3*) age of participants, *4*) design and intervention, *5*) type of DFS, *6*) iron concentration in DFS, *7*) details on what the control group received, *8*) DFS stability and organoleptic properties, *9*) coverage, *10*) average DFS intake, *11*) duration of intervention, *12*) safety concerns, *13*) baseline hemoglobin, ferritin, anemia, IDA, and morbidity, *14*) endline (i.e. the measurement of outcomes at the end of the study or the intervention) mean and SD for continuous outcomes and endline N values and prevalence for dichotomous outcomes, *15*) assessment tools, and *16*) quality ratings. Any discrepancies in data extraction between the reviewers were resolved with discussion and returning to the full-text articles. The analyses used outcome statistics measured at endline, immediately following the intervention.

Authors of studies with missing information were contacted twice requesting additional information. Two authors replied with the information requested.

DFS was categorized into types as presented by Nutrition International (15). Herein, Type 1a DFS refers to DFS that contains microencapsulated potassium iodide and ferrous fumarate; Type 1b contains encapsulated ferrous fumarate; Type 2 contains a refined iodized salt, ferrous sulfate, and a stabilizing compound; Type 3 contains ferrous sulfate with various chelating agents and encapsulated iodine; Type 4 contains encapsulated ferrous sulfate; and Type 5 contains micronized ferric pyrophosphate.

### Quality ratings

Quality was assessed and each study was assigned a global rating using the Effective Public Health Practice Project (EPHPP) quality assessment tool (17). The EPHPP assesses 8 dimensions: selection bias, study design, confounders, blinding, data collection methods, withdrawals and dropouts, intervention integrity, and robustness of the analysis. Each dimension is rated on a 3-point scale as strong, moderate, or weak, all of which contribute to the calculation of the global rating.

### Analyses

Statistical analyses were conducted in R 3.5.1 (R Foundation for Statistical Computing). Effect sizes for continuous outcomes were calculated using Hedge's g. Risk ratios were calculated for all dichotomous outcomes. Pooled analyses included randomized controlled efficacy trials. If studies were not randomized or if they were categorized as effectiveness trials, they were excluded from pooled calculations. Effect sizes and 95% CIs were calculated for urinary iodine and some ferritin concentrations from median and range values using a method presented by Hozo et al. 2005 (18). We assumed the median approximated the mean. Weights were assigned to each study by calculating the inverse variance of the endline scores. Pooled effect sizes and risk ratios for all outcomes were calculated by taking a weighted average of included studies (19). If studies reported outcomes for multiple population groups, a weighted average of all groups was used for the main analysis. Sensitivity analyses were conducted by running stratified pooled effect sizes and risk ratios by study quality, type of DFS [using Nutrition International classification as described above (15)], average iron intake from DFS (calculated by multiplying the iron concentration in DFS by average intake of DFS per person per day), baseline hemoglobin concentration, baseline ferritin concentration, baseline anemia prevalence, and intervention duration. Meta-regression was used to examine the association between effect sizes for hemoglobin and ferritin, with continuous baseline hemoglobin concentration, baseline ferritin concentration, anemia prevalence, intervention duration in months, and sample size. Significance was defined as *P *< 0.05.

Most studies examined a single type of DFS, with the exception of Andersson et al. (20), which reported effects using 2 intervention groups, 1 receiving DFS with micronized ferric pyrophosphate and another with encapsulated ferrous fumarate, both compared with the same control group. In our analysis, both intervention groups in this study were included as separate comparisons and the control group N was halved in pooled effect size calculations in order to avoid overweighting the study due to counting participants in the control group more than once.

If studies reported effects for 2 different populations with their own control group separately (e.g. children and women), both effects were included in the analysis as 2 separate intervention-control contrasts; thus, the N values reported represent the number of contrasts used in the analysis and may exceed the total number of studies.

Forest plots for each outcome were generated using the Metafor package in R 3.5.1. Statistical heterogeneity was assessed using the chi square test on the Cochrane's heterogeneity statistic Q and an *I^2^* statistic. Because of heterogeneity between studies, random effects models were used to create the pooled effect sizes and risk ratios. Publication bias was examined by generating funnel plots in R 3.5.1.

Lastly, we conducted an impact pathway review (21, 22), and created a program impact pathway, to guide an analysis of program implementation. We used studies collected from our systematic review to discuss fidelity of implementation and how evaluations published to date describe and agree with factors identified along our impact pathway and whether they were implemented in the way that they were intended to be (e.g. intensity of exposure, coverage, etc.).

## Results

### Study inclusion

**FIGURE 1:**
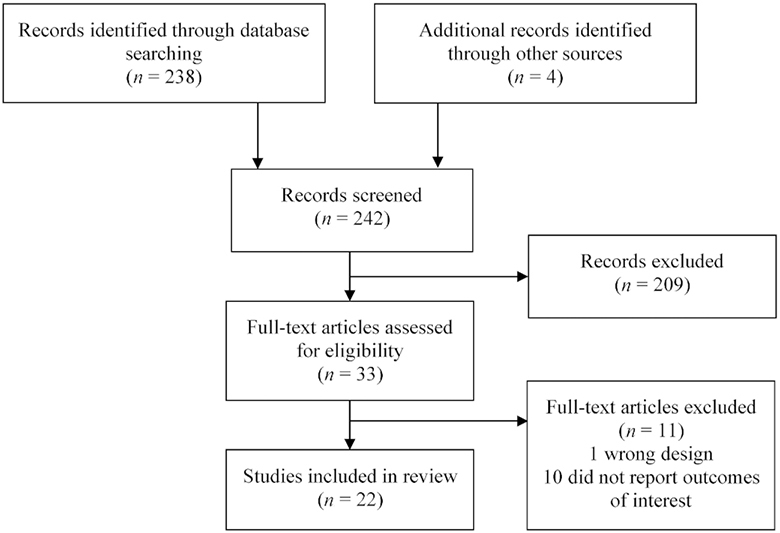
Study inclusion flow chart.

The database search strategy identified 238 records; 4 additional studies were identified through other sources (i.e. references from other articles) (**Figure 1**). Abstracts were screened for correct intervention, study design, and outcomes of interest, and identified 33 full-text articles for eligibility. A review of full-text articles excluded a further 11 articles that did not fit our inclusion criteria (1 because of study design, and 10 that did not measure any of the included outcomes). A total of 22 studies fitted our inclusion criteria, 8 of which were not included in the most recent published meta-analysis of DFS (14) (6 quasi-experimental designs; 2 nonpeer-reviewed papers) and 1 article recently published new outcome information from a previously reviewed study.

### Study characteristics

**FIGURE 2:**
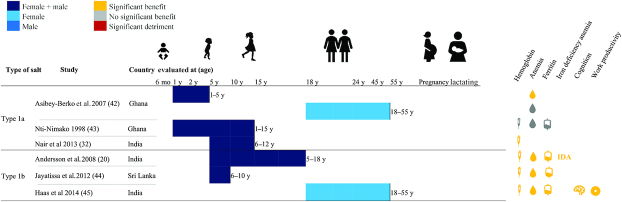
Summary of effects of double fortified salt on functional outcomes (DFS Types 1a and 1b). Significance of effects was determined by *P* values from analyses reported in the publications. Type 1a DFS refers to DFS that contains microencapsulated potassium iodide and ferrous fumarate; Type 1b contains encapsulated ferrous fumarate. DFS, double fortified salt; IDA, iron deficiency anemia.

**FIGURE 3:**
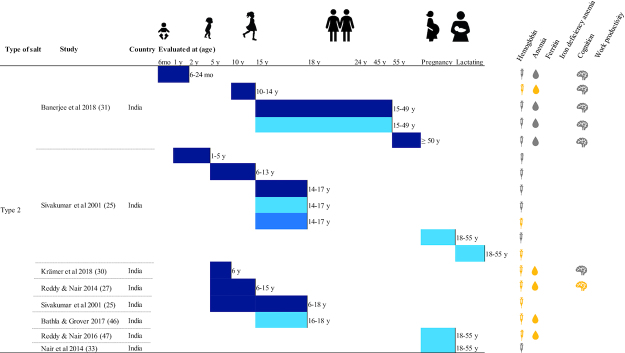
Summary of effects of double fortified salt on functional outcomes (DFS Type 2). Significance of effects was determined by *P* values from analyses reported in the publications. Type 2 contains a refined iodized salt, ferrous sulfate, and a stabilizing compound. DFS, double fortified salt; IDA, iron deficiency anemia.

**FIGURE 4:**
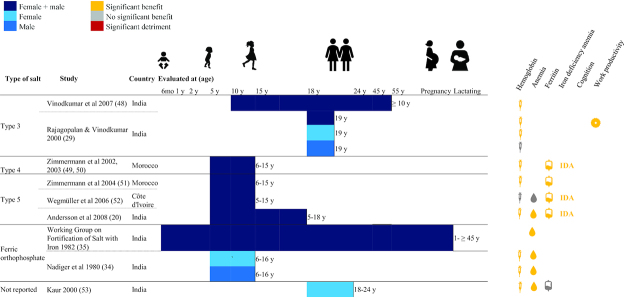
Summary of effects of double fortified salt on functional outcomes (DFS Types 3, 4, 5, and ferric orthophosphate). Significance of effects was determined by *P* values from analyses reported in the publications. Type 3 contains ferrous sulfate with various chelating agents and encapsulated iodine; Type 4 contains encapsulated ferrous sulfate; and Type 5 contains micronized ferric pyrophosphate. DFS, double fortified salt.

The majority of included DFS studies were conducted in India (N = 16); others were completed in Morocco (N = 2), Ghana (N = 2), Sri Lanka (N = 1), and Cȏte d'Ivoire (N = 1). Four studies examined children aged under 5 y, 16 studies examined school-age children (SAC) and adolescents (including 1 study that examined children aged 1–15 y), 3 studies examined adult women, 3 examined pregnant women, 1 examined lactating women, and 3 examined adult women and men. Different types of designs were observed: 16 reported results from efficacy trials, 2 from randomized effectiveness trials, 2 from nonrandomized designs, and 2 from pre-post designs. With the exception of 3 studies, all reported endline hemoglobin concentration, 8 reported ferritin concentration, 12 reported urinary iodine concentration, 14 reported anemia prevalence, 4 reported IDA prevalence, 4 reported various measures of cognition, and 2 reported work productivity using diverse measures (**Figures 2**–**4**, **Supplemental Tables 1** and **2**).

Many studies had a global quality rating of weak (N = 16); only 4 studies rated as moderate and 3 as strong. Importantly, although not accounted for in the quality rating, only 6 studies reported their power calculations or sample size estimation. Many studies lost points on their quality ratings due to selection bias, not accounting for confounders, and poor participant retainment.

### Outcome assessment methods

Assessment methods for the various outcomes are diverse (**Supplemental Table 3**). Depending on the study, hemoglobin concentration was measured using a range of methods including the cyanmethemoglobin method, Coulter counter, HemoCue, and Sahlil's method. Ferritin concentration was predominantly measured using ELISA, and urinary iodine concentration was typically measured using a modification of the Sandell-Kolthoff method.

### Publication bias

We do not suspect publication bias within efficacy studies. First, the funnel plots (**Supplemental Figures 1–6**) do not indicate that effect sizes are 1-sided. Second, smaller sample sizes were not significantly associated with larger effect sizes (23). Third, a roughly equal proportion of studies report effects above and below the standard level of statistical significance, which suggests that there is little risk of publication bias (24).

### Effects of DFS from efficacy trials

#### Hemoglobin

**FIGURE 5:**
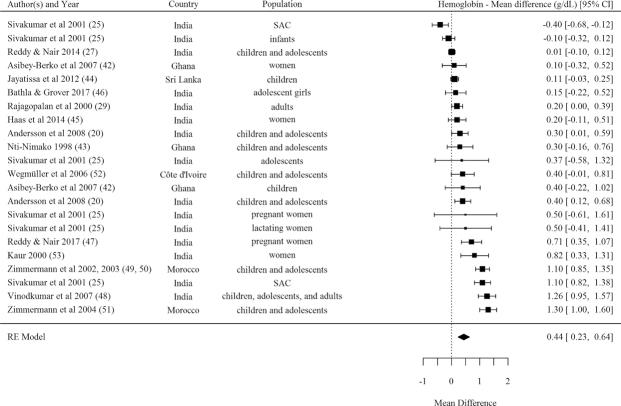
Forest plot for the effect of double fortified salt on hemoglobin concentration (mean difference). *I^2^* = 91.07%, Q(Df = 21)= 232.4953, *P *< 0.0001. SAC, school-age children; RE, random effects.

The pooled effect size indicated a significant effect from DFS efficacy trials on hemoglobin concentration, with a mean difference of 0.44 (95% CI: 0.23, 0.64) g/dL (N = 22 comparisons) (**Table 1**, **Figure 5**) and a standardized mean difference of 0.33 (95% CI: 0.18, 0.48) (**Supplemental Figure 7**). Effect sizes were stronger among studies rated as strong and moderate quality but were still significant among studies rated as weak quality. Effects on hemoglobin were significant for studies using DFS Type 1b (encapsulated ferrous fumarate) and Type 4 (encapsulated ferrous sulfate), and in those delivered to SAC and adolescents, nonpregnant adult women, and pregnant women (Table 1). Meta-regression estimates indicated no significant association between studies’ effect sizes for hemoglobin concentration and mean baseline hemoglobin, baseline anemia, baseline ferritin, sample size, or intervention duration in months.

**TABLE 1:** Pooled effect sizes and risk ratios for double fortified salt efficacy studies^1^

		Pooled effect sizes								Pooled risk ratios			
		N	Hemoglobin SMD	N	Hemoglobin MD (g/dL)	N	Ferritin SMD	N	Urinary iodine SMD	N	Anemia RR	N	Iron deficiency anemia RR
Overall		22	0.33 (0.18, 0.48)	22	0.44 (0.23, 0.64)	8	0.46 (0.21, 0.71)	7	–0.37 (–0.88, 0.13)	13	0.80 (0.70, 0.92)	5	0.36 (0.24, 0.55)
Quality													
	Strong or moderate	6	0.55 (0.18, 0.92)	6	0.62 (0.25, 1.00)	6	0.51 (0.19, 0.84)	4	–0.23 (–0.55, 0.09)	5	0.58 (0.40, 0.84)	5	0.36 (0.24, 0.55)
	Weak	16	0.23 (0.09, 0.38)	16	0.36 (0.12, 0.60)	2	0.34 (0.19, 0.50)	3	–0.56 (–1.77, 0.64)	8	0.91 (0.84, 0.99)	0	—
Type of DFS													
	1a	3	0.19 (–0.05, 0.43)	3	0.23 (–0.04, 0.51)	0	—	0	—	3	0.61 (0.27, 1.40)	0	—
	1b	3	0.18 (0.06, 0.31)	3	0.20 (0.03, 0.38)	3	0.37 (0.17, 0.57)	2	–0.05 (–0.52, 0.42)	3	0.73 (0.48, 1.12)	1	0.25 (0.10, 0.61)
	2	9	0.19 (–0.03, 0.41)	9	0.28 (–0.07, 0.63)	0	—	1	–1.78 (-1.95, -1.62)	3	0.95 (0.89, 1.01)	0	—
	3	2	0.34 (–0.05, 0.74)	2	0.72 (–0.32, 1.76)	0	—	1	-0.09 (–0.23, 0.04)	0	—	0	—
	4	1	0.91 (0.70, 1.13)	1	1.10 (0.85, 1.35)	1	1.17 (0.95, 1.39)	1	0.06 (–0.15, 0.26)	0	—	1	0.27 (0.16, 0.46)
	5	3	0.64 (–0.06, 1.34)	3	0.67 (0.04, 1.3)	3	0.39 (0.06, 0.73)	2	–0.34 (–0.96, 0.28)	3	0.54 (0.31, 0.93)	3	0.50 (0.32, 0.76)
Population	Infants	2	0.03 (–0.27, 0.32)	2	0.06 (–0.40, 0.52)	0	—	0	—	1	0.23 (0.05, 0.95)	0	—
	School-age children and adolescents	12	0.35 (0.11, 0.60)	12	0.43 (0.13, 0.73)	6	0.55 (0.26, 0.84)	6	–0.42 (–1.01, 0.17)	8	0.72 (0.55, 0.93)	5	0.36 (0.24, 0.55)
	Nonpregnant women	4	0.25 (0.07, 0.43)	4	0.30 (0.10, 0.51)	2	0.16 (–0.05, 0.37)	0	—	3	0.76 (0.63, 0.91)	0	—
	Pregnant women	2	0.59 (0.24, 0.95)	2	0.69 (0.35, 1.03)	0	—	0	—	1	0.91 (0.83, 1.01)	0	—
	Lactating women	1	0.30 (–0.21, 0.81)	1	0.50 (–0.41, 1.41)	0	—	0	—	0	—	0	—
	Men	1	0.08 (–0.14, 0.30)	1	0.12 (–0.21, 0.45)	0	—	0	—	0	—	0	—
Average iron intake from DFS	≤10 mg iron/(person·d)	12	0.27 (0.09, 0.46)	12	0.45 (0.13, 0.76)	1	0.18 (–0.16, 0.52)	2	–0.94 (–2.59, 0.72)	4	0.79 (0.58, 1.08)	0	—
	>10 mg iron/(person·d)	4	0.44 (0.09, 0.79)	4	0.52 (0.10, 0.93)	6	0.51 (0.19, 0.84)	2	–0.12(–0.46, 0.23)	3	0.59 (0.25, 1.37)	5	0.36 (0.24, 0.55)
Baseline hemoglobin concentration	<11 g/dL	10	0.23 (0.02, 0.43)	10	0.34 (0.00, 0.67)	1	0.18 (–0.16, 0.52)	1	–0.09 (–0.23, 0.04)	4	0.91 (0.83, 1.00)	0	—
	≥11 g/dL	11	0.41 (0.18, 0.65)	11	0.53 (0.26, 0.81)	6	0.56 (0.29, 0.84)	6	–0.42 (–1.01, 0.17)	8	0.61 (0.43, 0.87)	5	0.36 (0.24, 0.55)
Baseline anemia prevalence	≤50%	6	0.28 (0.12, 0.44)	6	0.40 (0.09, 0.72)	3	0.46 (0.34, 0.59)	4	–0.64 (–1.46, 0.19)	5	0.63 (0.38, 1.06)	2	0.34 (0.20, 0.60)
	>50%	8	0.46 (0.15, 0.76)	8	0.48 (0.17, 0.79)	4	0.23 (0.00, 0.45)	1	–0.02 (–0.33, 0.30)	8	0.84 (0.74, 0.95)	2	0.43 (0.13, 1.38)
Intervention duration	<12 months	14	0.40 (0.20, 0.60)	14	0.45 (0.23, 0.66)	8	0.46 (0.21, 0.71)	5	–0.14 (–0.44, 0.16)	13	0.80 (0.70, 0.92)	5	0.36 (0.24, 0.55)
	≥12 months	8	0.20 (–0.02, 0.43)	8	0.42 (–0.03, 0.87)	0	—	2	–0.94 (–2.59, 0.72)	0	—	0	—

1 N represents the number of intervention-control contrasts. Values are effect sizes or risk ratios (95% CI). Urinary iodine effect sizes include only studies where the intervention group received DFS and the control group received iodized salt. Type 1a DFS refers to DFS that contains microencapsulated potassium iodide and ferrous fumarate; Type 1b contains encapsulated ferrous fumarate; Type 2 contains a refined iodized salt, ferrous sulfate, and a stabilizing compound; Type 3 contains ferrous sulfate with various chelating agents and encapsulated iodine; Type 4 contains encapsulated ferrous sulfate; and Type 5 contains micronized ferric pyrophosphate. Studies using Type 5 DFS were excluded from the stratified analyses by average iron intake from DFS because the lower bioavailability of iron from this type of DFS makes it noncomparable to the other types of salts. DFS, double fortified salt; MD, mean difference; RR, risk ratio; SMD, standardized mean difference.

#### Ferritin

**FIGURE 6:**
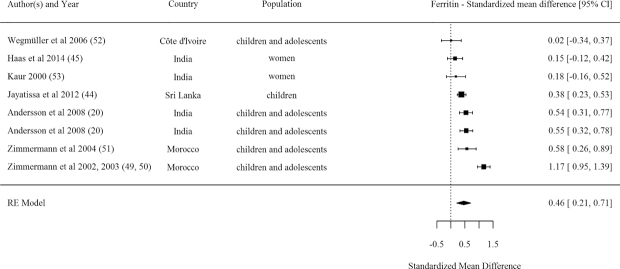
Forest plot for the effect of double fortified salt on ferritin concentration (standardized mean difference). *I^2^* = 87.83%, Q(Df = 7)= 55.0956, *P *< 0.0001. RE, random effects.

A pooled effect size of results from 8 efficacy comparisons showed a significant positive effect from DFS on ferritin concentration (Table 1, **Figure 6**). Most of these studies were conducted in SAC and adolescents. The majority of studies measuring ferritin (i.e. 6 out of 8) rated as strong or moderate quality. Three out of the 8 comparisons examined DFS Type 1b (encapsulated ferrous fumarate) and 3 others examined Type 5 (micronized ferric pyrophosphate)—both DFS types found positive effects on ferritin. The meta-regression analysis indicated no significant association between studies’ effect sizes for ferritin concentration and mean baseline hemoglobin, baseline anemia, baseline ferritin, sample size, or intervention duration in months.

#### Urinary iodine

Compared with iodized salt, DFS did not affect urinary iodine concentrations in pooled analyses of 7 efficacy studies (Table 1, **Supplemental Figure 8**). However, 2 studies demonstrated significant negative effects. In India, Sivakumar et al. conducted a randomized controlled trial in 4 residential schools, randomized to receive either Type 2 DFS (ferrous sulfate + stabilizing compound) or iodized salt (25). They reported that the iodine content of the DFS was below acceptable limits and hypothesized this was due to poor quality control at the production level and bulk packaging of the DFS. In another study by Andersson et al., children and adolescents received either Type 5 DFS (micronized ferric pyrophosphate), Type 1b DFS (encapsulated ferrous fumarate), or iodized salt (20). Type 1b DFS did not report differences in iodine content compared with iodized salt after 6 mo (both salts lost 20% of their iodine content), whereas iodine losses in the Type 5 DFS were 44% over the first month of storage and 86% over 6 mo.

#### Anemia

**FIGURE 7:**
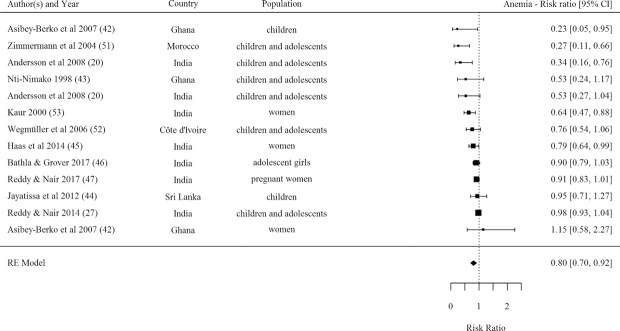
Forest plot for the effect of double fortified salt on anemia (risk ratio). *I^2^* = 71.88%, Q(Df = 12)= 35.2512, *P* = 0.0004. RE, random effects.

Effects on anemia prevalence were observed from a pooled analysis of 13 DFS efficacy studies (Table 1, **Figure 7**). Overall, those receiving DFS had 0.80 times the risk of anemia than controls. Evidence for reduced risk of anemia was larger in strong- and moderate-quality studies compared with weak-quality studies. Pooled effects stratified by type of DFS indicated significant effects from Type 5 DFS (micronized ferric pyrophosphate) only. The majority of studies reporting effects on anemia were conducted in SAC and adolescents and in pregnant women and effects were significant in both populations.

#### IDA

**FIGURE 8:**
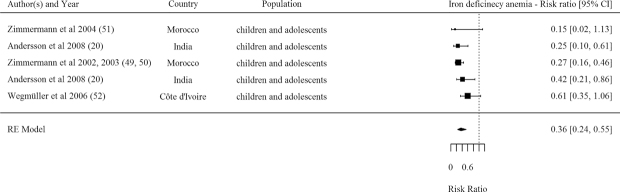
Forest plot for the effect of double fortified salt on iron deficiency anemia (risk ratio). *I^2^* = 57.31%, Q(Df = 4) = 9.5192, *P* = 0.0494. RE, random effects.

Only 5 studies reported effects on IDA and overall the pooled effect size indicates that those receiving DFS had 0.36 times the risk of IDA than controls (Table 1, **Figure 8**). All included studies were rated as strong or moderate.

#### Cognition

Significant positive effects on cognitive development were reported in 2 efficacy trials, however, assessment measures were too different to pool results. In Haas et al.’s study of adult women tea pickers in India, positive effects were observed on perception, attention, and memory (26). In another school-based study of Indian children and adolescents, significant effects were observed on memory and cognition (27). No study reported effects of DFS on child mental or motor development.

#### Work productivity

Only 2 efficacy studies measured effects of DFS on work productivity. In Indian female tea pickers, effects were observed on work output in terms of leaves picked (28). In another trial in adult female and male tea pickers in India, the average daily quantity of tea leaves picked by an individual increased in those who received DFS and had been dewormed compared with controls (29).

No study reported effects of DFS on morbidity.

#### Effects of DFS from effectiveness trials

Results from efficacy trials indicate that under controlled conditions, DFS has significant effects on hemoglobin, ferritin concentration, anemia, and IDA prevalence. Effectiveness trials demonstrate programmatic effects, when DFS is distributed through diverse programmatic platforms.

Two effectiveness studies reported results from DFS distributed through existing channels in India. Kramer et al.’s trial in school children randomly assigned 54 schools to distribute Type 2 DFS (ferrous sulfate + stabilizing compound)-fortified food through the midday meal school feeding program and 54 schools to distribute regular salt (30). Coverage statistics were not reported but authors report that it was likely close to 100% because few shortages were reported, and monitoring visits indicated that schools were using DFS in the midday meal program. Results from 2812 children indicated significant effects on hemoglobin concentration (β = 0.136, SE = 0.076, *P* value < 0.10) and anemia prevalence (β = 0.093, SE = 0.033, *P* value < 0.01), but not cognition and education results (math and reading test results, and school attendance).

Banerjee et al.’s study in Indian children, adolescents, and adults included 2 randomized allocations, 1 nested within the other (31). The first experiment sold Type 2 DFS (ferrous sulfate + stabilizing compound) at a reduced price through private village shops and the Public Distribution System, a food security system that provides staple foods at subsidized rates to lower income families. Stability and organoleptic properties were not reported. Coverage indicators demonstrated that 42.5% of households in villages where DFS was sold had ever tried DFS, and only 14.5% of households reported using DFS at the end of the study. Within this experiment, some villages received information campaigns. In villages where an educational movie was shown and in villages where shopkeepers were given an incentive to market DFS, uptake of DFS was 5 percentage points higher. No significant effects were observed on hemoglobin concentration, cognition, physical fitness, or mental health overall; significant effects were seen on hemoglobin and anemia when examining adolescents separately. The second experiment was nested within the first experiment. In a subset of 62 villages that were allocated to the DFS group, the same DFS was distributed to a random subset of households free of cost. Coverage was higher in this experiment, in households with free distribution; 61% of households were using DFS at the time of the survey, and an additional 14% of households had been using it and had only recently run out. Despite the higher coverage, no significant effects were seen on hemoglobin, anemia, cognition, physical fitness, or mental health, overall and by population group. No indicators of iron status or IDA were included as primary or secondary outcomes.

Two studies used a pre-post design only following up those who received DFS. One study in 3125 Indian children with goiter or low urinary iodine found significant improvements in hemoglobin concentration in those receiving Type 1a DFS (microencapsulated potassium iodide + ferrous fumarate) (32). In another study of critically anemic pregnant women, no significant change in hemoglobin was observed in women receiving Type 2 DFS (33). Two other studies used nonrandomized designs and groups were not comparable at baseline (34, 35).

#### Impact pathway analysis

**FIGURE 9:**
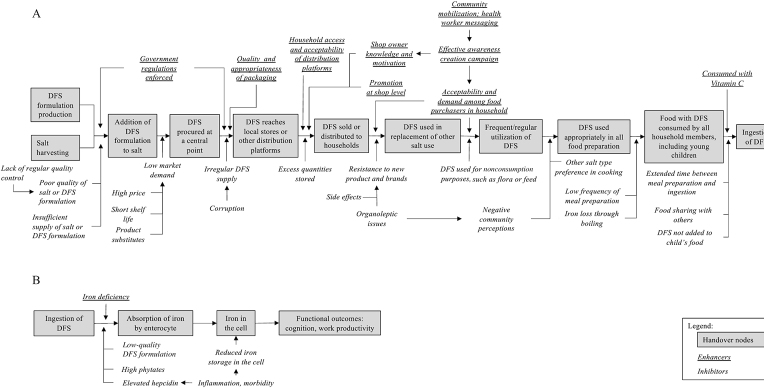
Double fortified salt program impact pathway. Panel A presents pre-ingestion program impact pathway and Panel B presents post-ingestion program impact pathway. DFS, double fortified salt.

To fully interpret the impacts of efficacy and effectiveness trials and assess internal and external validity, it is essential to understand the context of implementation and the impact pathways (36). To aid in our impact pathway review, we created a program impact pathway for DFS programs specifically (**Figure 9**). Discussions of factors along the impact pathway remain limited in the studies published to date and are important to address in order to guide effective DFS programs (37)—factors related to the product itself, the quality and potential of the platform to deliver that supply, and the extent to which the potential users are made aware and motivated/empowered to acquire and use DFS. In terms of the product, the process of DFS formulation production can have several bottlenecks; many factors, such as price of ingredients, insufficient use of certain components during formulation production due to cost-cutting measures adopted by the manufacturers, lack of stringent quality control measures to ensure standardized formulation production, can affect the quality of the formulation produced, which in turn can cause color changes in the final DFS product or when used in cooking (38). Several supply related issues (transportation and storage challenges, intentionally and unintentionally providing DFS to those who should not be receiving it) may similarly affect distribution and can, if not identified and resolved, result in inequities that can prevent some of those in need from accessing stores that are selling or points of distribution of DFS. Demand creation for DFS can be a fundamental requisite for program success in contexts where mandatory legislation is not present. The studies we reviewed were mostly efficacy trials in controlled settings where DFS was the only salt provided to the treatment group—and most studies included activities to promote consumption. In a programmatic setting, particularly those with for-purchase DFS, DFS may fail to effectively substitute for alternative salt types if there is not an effective awareness creation campaign that informs consumers about its benefits or unless a product of adequate quality is provided at such a favorable price. Finally, cooking patterns (i.e. timing of salt addition, boiling duration, addition of salt with cooking or after) and feeding patterns (i.e. addition of salt to children's meals) or eating behaviors (i.e. how long after food preparation is the meal consumed) can affect the stability of the iron and thus appearance of the food. This in turn may influence a consumer's decision to continue DFS use (37). An example of some of these challenges observed through a DFS program evaluation in Uttar Pradesh, India, is described in **TextBox1**. This topic is more comprehensively covered in the third paper in this supplement. It is important to consider these aspects and identify solutions that can address some of these challenges as we go on to implement and scale-up DFS programs.

Text Box 1: DFS program in Uttar Pradesh, India

Distribution of DFS took place through the fair price shops in the Public Distribution System (PDS), which also sold rice, wheat, and kerosene at subsidized prices to eligible households. The program achieved high coverage, with over 70% of households having ever purchased DFS. However, some constraints pertaining to perceived quality, and organoleptic changes, were also noted.

Although ∼82% of respondents considered the quality of the grains and kerosene to be “good,” only ∼32% of respondents in households considered the DFS to be “good” quality at midline (i.e. the measurement of outcomes at midway of the intervention). Additionally, most households had purchased DFS (as the PDS often does a bundled sale of grains, kerosene, and salt), but only a third admitted to typically using DFS in preparing all family meals. Awareness about the health benefits of DFS was poor, and qualitative data confirmed that few caregivers had knowledge about iron being present in DFS. Compromises made by salt producers during DFS production— by using poor quality encapsulation, with little or no white coating (titanium dioxide) for the ferrous fumarate in the DFS formulation—led to clearly identifiable black “specks” in the salt and reactivity with food. This was reflected in several caregivers complaining about dark specks in DFS, food turning darker etc. A few respondents thought these were tiny stones present in the salt. One caregiver mixed DFS in a glass of water and reported seeing “*something like ash*” accumulating at the bottom. Most caregivers found no change in taste and selectively used DFS depending on the dish that was prepared. DFS was used when dark leafy green vegetable dishes (locally known as *saag)* were prepared and avoided in lighter colored dishes like yellow lentils (locally known as *dal*)— “*the blackness is visible only in those dishes in which we use turmeric, whereas in green saag vegetables the DFS color makes no difference*.”

## Discussion

This review of randomized efficacy studies and 2 program effectiveness evaluations demonstrates the potential for DFS to improve iron status across several population groups. The evidence of biological potential (i.e. efficacy studies) is strong and consistent for an increase in hemoglobin and ferritin concentrations, and reduction in anemia and IDA prevalence. The magnitude of that impact varied, particularly for hemoglobin concentration, with larger impact for those using Type 1b (encapsulated ferrous fumarate) and Type 4 DFS (encapsulated ferrous sulfate), among SAC and adolescents, nonpregnant women, and pregnant women.

Only 2 program effectiveness evaluations were identified and included, both providing DFS in India. One study of DFS distributed through school feeding programs with high coverage, found a significant increase in hemoglobin concentration and reduction of anemia prevalence. The other study of DFS sold through local markets and ration shops only observed significant effects on hemoglobin and anemia in adolescents, but not infants or adult men and women. Based on our review of design and implementation considerations we identified several factors that may favor potential for impact of DFS.

### Considerations for achieving the biological potential of DFS

The iron requirements and gap from the diet of populations from different settings will dictate their response to an iron intervention. Several studies reviewed here included exclusively anemic or iron-deficient populations, whereas others were implemented in populations with moderate or low prevalence of ID/IDA. Theoretically, populations with higher ID have greater potential to benefit, as long as the iron consumed from DFS is sufficient. We found a statistically nonsignificant tendency to greater impact in studies where baseline anemia prevalence was >50% compared with <50%, and when the mean iron intake from DFS was >10 mg/(person·day). But few studies were available to confirm this tendency. No significant association was found between study effect size and intervention duration, likely because changes in hemoglobin and ferritin concentrations occur relatively quickly after supplementation with iron. The majority of studies intervened for 3 mo or more, which is sufficient to observe a rise in these biomarkers (8).

Pooled effect sizes for DFS efficacy trials indicated that those receiving DFS had 0.44 (95% CI: 0.23, 0.64) g/dL higher hemoglobin concentrations than controls and 12.7 (95% CI: 5.8, 19.6) μg/L higher ferritin concentrations. Effects from other food fortification interventions with multiple micronutrients (7) have shown larger effects, which may be explained by the low iron content of DFS or by the varied etiology of anemia in the various study populations.

Biological effects of DFS vary by formulation. Overall, Type 1b (encapsulated ferrous fumarate) and Type 4 (encapsulated ferrous sulfate) DFS indicated significant positive effects on hemoglobin, although only 1 study contributed to effects of Type 4 DFS. Type 1b, Type 4 (only 1 study), and Type 5 DFS (micronized ferric pyrophosphate) resulted in positive effects on ferritin. The type of DFS overlaps somewhat with the quality of studies, wherein the majority of studies using Type 1b, Type 4, and Type 5 DFS were rated as moderate or high quality. At this time, we cannot disaggregate the potential implications of study quality compared with DFS type.

### Considerations for programmatic potential

Based on the above results, in populations with ID where programs select an appropriate type of DFS, the potential for impact is high. DFS programs should be considered only in contexts with evidence of inadequate dietary iron intake/deficiency. However, several programmatic factors may still limit or favor the realization of this potential. Based on existing evidence (37), demand creation is an important challenge experienced by several programs. Salt is a ubiquitous low-cost commodity in most settings and options other than DFS will be available in markets. Thus, regardless of program design, including free distribution or not, quality demand creation is needed to foster high coverage, utilization, and ultimately biological impact. Quality demand creation can take many forms, but must be responsive to local preferences, traditions, and needs (37). Where DFS is distributed or sold, there may be advantages if shop keepers or others are able to describe the benefits of DFS to customers and answer basic questions. As seen in the study by Banerjee et al. (31) in India, coverage increased in areas where there was social marketing.

The consistent quality assurance of DFS is another aspect that requires close monitoring and rapid response to address identified issues. At the factory level, DFS formulation quality has been shown to vary substantially (39), with important downstream effects on iodine content and on biological potential. Further, poor quality control at the production, warehouse, and sale level influences the quality of DFS at the time it reaches the household, color changes in foods cooked with DFS, and ultimately its acceptability and utilization. Program evaluations reviewed did not adequately document potential bottlenecks and issues related to DFS quality and distribution across a clear pathway to impact; such information is critical to inform timely course correction in programs but also to support the interpretation of impact evaluation findings. A null result without such information to confirm the extent of program rollout and quality contributes little to the evidence base.

### Implications of DFS for iodine and salt iodization

Salt iodization is an impactful program implemented at scale in many countries globally (40). Whether iodine is lost in DFS to a greater extent than iodized salt is therefore an important programmatic consideration. Most, but not all, trials used a control or comparison group which received iodized salt; others received no intervention. In this review, pooled analyses for effects on urinary iodine were limited to trials where the control group received iodized salt. No significant effects were observed on urinary iodine indicating that, overall, those consuming DFS do not have lower iodine status than those consuming iodized salt. That said, some studies reported significant iodine losses in their DFS, specifically with DFS Type 2 (ferrous sulfate + stabilizing compound) and 5 (micronized ferric pyrophosphate). In these few studies, significant differences in iodine status between groups receiving DFS and those receiving iodized salt were not because the iodine in DFS had a reduced biological effect post-ingestion. Instead, differences in iodine levels by treatment group were largely due to poor quality control at the production level and suboptimal storage conditions, which led to reduced iodine in the DFS preconsumption. Iodine and its potential loss in DFS are further discussed elsewhere (16).

### Considerations for safety

Iron is an essential element in living organisms but can pose risks when in excess and during times of infection. Excess unabsorbed iron progresses to the colon where it has the opportunity to interact with gut microbiota and may result in increased inflammation and diarrheal risk (10). Iron can also feed parasites and increase the risk of malaria (11, 12). Therefore, supplementing individuals or populations with iron, particularly if they are already iron replete, may lead to higher risk of infection and morbidity. However, these concerns are most relevant to interventions using high-dose iron supplements or high-iron-containing MNPs (10–12). The iron concentration contained in DFS is several-fold lower than that contained in the majority of iron tablets and MNPs (i.e. 1 mg/kg salt in DFS compared with 10–12.5 mg/sachet in MNPs). The risk of adverse morbidity effects therefore should be substantially lower. None of the studies included in this review reported effects of DFS on morbidity from iron. Although the risk for increased morbidity is low, future efficacy studies should consider monitoring increased diarrhea, respiratory infections, malaria, and hospitalizations. Although information on morbidity should be added to future efficacy research, given the biological effects of DFS outlined in this review and the relatively low concentration of iron in DFS, it is highly likely that the benefits outweigh the risks.

### Quality of the evidence

Internal validity of the efficacy trials was generally weak, with only 6 out of 22 studies rated as moderate or high quality. Primarily, RCTs included in this review had low internal validity because of issues around selection bias, unaccounted confounders, and participant withdrawals. Trials that reported significant effects on hemoglobin and ferritin concentrations were those that reported low dropout (<20%) and high intervention integrity (i.e. high coverage and consistency of the intervention). The external validity of DFS effectiveness trials also lacks strength due to low coverage and integrity of the DFS; several of the evaluations included here did not provide sufficient information to assess the quality of implementation, coverage, or utilization of DFS. Finally, there are several product development issues that require further study and attention within programs. First, ensuring high quality and continual quality control and assurance for DFS at the production level. Second, exploring whether product stability can be improved, and as a minimum, consumer education strengthened to overcome product alteration due to inadequate storage in point of sale/distribution and in the home.

### Limitations

This review includes several analytic limitations. We compared endline values for the outcomes of interest, assuming that baseline values will be comparable across intervention groups or adjusted for in the analysis. However, a minority of studies do not adjust for baseline values or confounders despite there being important differences between intervention groups, which should be accounted for in the analysis. In order to differentiate between the types of studies, we presented pooled effect sizes stratified by study quality to demonstrate effects for studies of high- and moderate-quality compared with weak quality. Too few studies reported change from baseline to endline to pool this data. As in any meta-analysis, we pooled study-level data, and used study-level means to create stratified pooled effect sizes. The use of aggregated study-level data may be masking associations observed at the individual level.

Indicator choice to assess impact of DFS was an important limitation of the identified studies. Most studies reported effects on hemoglobin concentration and/or anemia prevalence. The limitations of hemoglobin as a sole indicator of iron interventions is now well recognized due to the multiple etiology of anemia as discussed previously. Few of the identified studies assessed impact on iron status (serum ferritin or other biomarkers), and of those that did, many did not adequately adjust for inflammation (41), which elevates ferritin concentration (41). Failing to adjust ferritin for inflammation may not have influenced the differential impact of DFS compared with control (assuming no systematic difference in the prevalence of inflammation between groups), but will affect the accuracy of ID prevalence estimates. Evaluations and studies of DFS and other iron interventions should focus on indicators of iron status and IDA, using appropriate adjustments for infection and inflammation.

We caution readers on overinterpretation of the stratified analyses. Too few studies exist to make strong conclusions about the superiority of 1 strata over another. For instance, we would have preferred to run more nuanced analyses to examine differences in outcomes by the amount of iron consumed through DFS; however, the number of studies reporting consumption of salt limited this possibility.

### Further research needs

Although effects from Type 4 DFS (encapsulated ferrous sulfate) are significant, only 1 study contributed to those findings on hemoglobin, anemia, and IDA, and further evidence is required to determine the optimal iron component for DFS. Only 2 effectiveness studies have been published to date. Future impact evaluations should use the most robust designs and always include a clear intended pathway to impact and ensure ample data including quality, distribution, coverage, and utilization, among other intermediate outcomes, to support the interpretation of conclusions. Importantly, these data should be used in real-time to correct implementation issues—as is good practice for all programs.

### Conclusions

Overall, DFS was found to be efficacious to improve hemoglobin concentration, and to reduce the prevalence of anemia and IDA among several population groups across several settings. We found no evidence that DFS affects iodine status differently from iodized salt; when iodine losses were reported, they were presumed to be due to suboptimal quality control and storage. Limited effectiveness research has indicated important implementation issues, particularly with respect to consistent quality of DFS, demand creation, coverage, and utilization, but poor evaluation design may have limited potential to detect impact. Close attention should be paid to ensure robust design of future impact evaluations, and foster good practice of continual course correction of identified design and implementation issues.

## Supplementary Material

nxaa192_Supplemental_File

## Notes

Funding for this review was obtained from the Iodine Global Network.

Author disclosures: The authors report no conflicts of interest.

Published in a supplement to *The Journal of Nutrition*. The Guest Editor for this supplement was Becky Tsang and she has no conflicts of interest. Publication costs for this supplement were defrayed in part by the payment of page charges by the Iodine Global Network. The opinions expressed in this publication are those of the authors and are not attributable to the sponsors or the publisher, Editor, or Editorial Board of *The Journal of Nutrition*.

The Supplement Coordinator for the supplement publication was Becky Tsang, Iodine Global Network, Seattle, WA, USA. She reports no conflicts of interest.

Supplemental Tables 1–3 and Supplemental Figures 1–8 are available from the ‘‘Supplementary data’’ link in the online posting of the article and from the same link in the online table of contents at https://academic.oup.com/jn/.

Abbreviations used: DFS, double fortified salt; EPHPP, Effective Public Health Practice Project; ID, iron deficiency; IDA, iron deficiency anemia; MNP, multiple micronutrient powder; SAC, school-age children.
