# Supplementary material for: Can Double Fortification of Salt with Iron and Iodine Reduce Anemia, Iron Deficiency Anemia, Iron Deficiency, Iodine Deficiency, and Functional Outcomes? Evidence of Efficacy, Effectiveness, and Safety
Source: J Nutr. 2021 Feb 15;151(Suppl 1):15S–28S. doi: 10.1093/jn/nxaa192 (PMC7882357; doi:10.1093/jn/nxaa192)
Supplement: nxaa192_Supplemental_File [file nxaa192_supplemental_file.docx]

Title: Can double fortification of salt with iron and iodine reduce anemia, iron deficiency anemia, iron deficiency, iodine deficiency, and functional outcomes? Evidence of efficacy, effectiveness, and safety

First author: Leila M Larson

Online Supplementary Material

Supplemental Table 1: Summary of double fortified salt study characteristics

| **Author and year** | **Country** | **Design and intervention** | **DFS type and iron concentration** | **DFS stability and organoleptic properties** | **Iodine loss from storage** |
| --- | --- | --- | --- | --- | --- |
| Asibey-Berko et al 2007(42) | Ghana | Double blind randomized comtrolled efficacy trial in agrarian region of Sekyere West district of Ghana in which mildly anemic or non-anemic women and their mildly anemic and non anemic children were enrolled and received either DFS or iodized salt. Duration 8 months. | Type 1a;  1mg iron/kg DFS | DFS found to be stable after 12 months of storage. The DFS was slightly darker in color compared to iodised salt. 2.7% of women in the DFS group reported darkening of plantain when fried with DFS. Food taste did not change and remained acceptable. | - |
|  |  |  |  |  |  |
| Nti-Nimako 1998(43) | Ghana | Double blind randomized controlled efficay study in children with Hb>=10 g/dL from 9 communities in the Sekyere west district who received DFS or iodized salt. Duration 4 months. | Type 1a;  1mg iron/kg DFS | No differences in taste, but lower appearance and acceptability for DFS. | - |
| Nair et al 2013(32) | India | Pre-post design study in 11 randomly selected villages in rural Vadodara. 3125 children examined for goitre and urinary iodine to determine eligibility. Included only children with goiter and low urinary iodine excretion. Only intervention children followed up. Duration 2 months. | Type 1a;  1mg iron/kg DFS | - | - |
| Andersson et al 2008(20) | India | Double blind randomized controlled efficacy trial in 18 villages in Bangalore, children recruited from 6 schools, 5 primary schools, and 1 high school and randomized to DFS or iodized salt. Duration 10 months. | Type 5;  2 mg iron/kg DFS | Negligible color difference between DFS (light beige) and iodized salt. No detectable difference in color, odor, or taste. Rated as acceptable. | Iodine stability testing was performed on the 2 different types of native salts used in the trial, salt with 0.5% moisture and with 1.8% moisture; the IS, MGFePP, and EFF salts of the 2 different qualities were locally stored as 2.5kg batches in closed, high-density, transparent polyethylene bags that were stored indoors under local ambient conditions and out of direct sunlight. Iodine losses in the MGFePP salt were 44% over the first month of storage and 86% over 6 m of storage in salt with 1.8% moisture. |
|  |  |  | Type 1b;  2 mg iron/kg DFS | Slight color difference between DFS (light grey) and iodized salt. When added to the water used in cooking rice, EFF produced small black spots on the surface of the cooked rice grains. Rated as acceptable. | There was no difference in iodine stability between the EFF and the IS: both salts lost 20% of their iodine content after 6 m. |
| Jayatissa et al 2012(44) | Sri Lanka | Cluster randomized community efficacy trial in rural and urban areas of Gampaha in which communities were randomly assigned to receive DFS or iodized salt. Duration 9 months. | Type 1b;  1 mg iron/kg DFS | The main problem identified relevant to low acceptability of DFS was change in colour and this was identified as a decisive factor while purchasing salt. 5.9% of households in the DFS group, compared to 2.6 % in the iodine group, complained about the color of the salt. | - |
| Haas et al 2014(45) | India | Double blind randomized controlled efficacy trial in healthy non-pregnant women who worked as full time tea pickers in Darjeeling district of India and randomized to DFS or iodized salt. Albendazole was administered to all eligible participants 4 weeks before baseline and at midline. Duration 9 months. | Type 1b;  1.1 mg iron/kg DFS | - | - |
| Banerjee et al 2018(31) | India | First experiment made DFS available in shops and Public Distribution System at a reduced price (9 rupees) in 200 randomly selected villages in Bihar. Control group received nothing (DFS was not available for sale). Duration 23 months. | Type 2;  1mg iron/kg DFS | - | - |
|  |  |  |  |  |  |
|  |  |  |  |  |  |
|  |  |  |  |  |  |
|  |  |  |  |  |  |
|  |  |  |  |  |  |
|  |  | Second experiment was embedded in the first sales experiment. In 62 villages where DFS was being sold in shops, a regular supply of free DFS was distributed to a random subset of homes. Duration 23 months. | Type 2;  1mg iron/kg DFS | - | - |
|  |  |  |  |  |  |
|  |  |  |  |  |  |
|  |  |  |  |  |  |
|  |  |  |  |  |  |
|  |  |  |  |  |  |
| Sivakumar et al 2001(25) | India | Unblinded randomized efficacy trial where four blocks in villages in the tribal areas of East Godavari district (Andhra Pradesh) were randomly assigned to receive either DFS, iron-fortified salt, iodized salt, or unfortified salt. Here, we only present DFS vs unfortified salt findings. Cross-sectional samples of individuals were tested at baseline and endline (not necessarily following up same individuals). Duration 24 months. | Type 2;  1mg iron/kg DFS | Acceptability of DFS was tested by assessing knowledge, attitude, and practices. No complaints of any side effects on consumption of DFS. | The stability of iodine in DFS, when tested by spot test every 3 months under the operational conditions prevailing at these tribal households, was more than 15 ppm and therefore at an acceptable level. |
|  |  |  |  |  |  |
|  |  |  |  |  |  |
|  |  |  |  |  |  |
|  |  |  |  |  |  |
|  |  |  |  |  |  |
|  |  |  |  |  |  |
|  |  |  |  |  |  |
| Kramer et al 2018(30) | India | Randomized effectiveness trial in second grade children in Bihar assigned to receive DFS-fortified food through the mid-day meal school feeding program. 54 schools were assigned to receive the DFS at a subsidized price, and the 54 control schools received regular salt. Duration 12 months. | Type 2;  1mg iron/kg DFS | Not described, but laboratory studies have shown good stability of the iron and iodine content of this DFS formulation. | - |
| Reddy & Nair 2014(27) | India | Cluster randomized efficacy trial in school children from rural villages of Vadodara, Gujarat. 2 schools were assigned to DFS intervention and 2 schools to control group (received nothing, but recommended to continue consuming iodized salt). Deworming given to half the intervention and half the control children. Duration 9 months. | Type 2;  1mg iron/kg DFS | - | - |
| Sivakumar et al 2001(25) | India | Double blind randomized controlled efficacy trial where four residential schools were randomized to receive either DFS or iodized salt. Duration 24 months. | Type 2;  1mg iron/kg DFS | - | The iodine content of DFS collected from the residential schools was below acceptable limits (<15 ppm) in the first and the third batch; two reasons cited for this potential iodine loss in DFS were 1) salt supplier not maintaining good quality control at the production level (magnesium and insoluble solid content particularly exceeded prescribed limits) and 2) the bulk packing of the fortified salt supplied for the school (50kg packets) as against 1kg packets of iodine (used in the tribal households study). |
| Bathla & Grover 2017(46) | India | Efficacy study in anemic adolescents attending government schools in Ludhiana District (Punjab) who received DFS or iodized salt. No description of random allocation or if random design. Duration 3 months. | Type 2;  1mg iron/kg DFS | - | - |
| Reddy & Nair 2016(47) | India | Randomized efficacy trial in pregnant women recruited in their first trimester from a semi government hospital of urban Vadodara, Gujarat, to receive DFS or nothing (but recommended to continue consuming iodized salt). Duration 6 months. | Type 2;  1mg iron/kg DFS | - | The stability of DFS (with mean iodine content 40ppm and mean iron content 1050ppm) was assessed after 1 year; the contents remained close to the recommended levels for iodine (37.5 ppm) and iron (979 ppm). In this study, iodine in DFS was more stable and provided more iodine compared to iodized salt. |
| Nair et al 2014(33) | India | Pre-post design study in critically anemic pregnant women living in tribal areas of Jhagadia. No comparison group. Women supplemented with DFS and iron and folic acid. Duration 2 months. | Type 2;  1mg iron/kg DFS | - | - |
| Vinodkumar et al 2007(48) | India | Single blind randomized efficacy trial in 7 centers in 3 states in India, where individuals were randomized to receive either DFS or iodized salt. All participants dewormed at baseline, after 6 months, and at endline. Duration 12 months. | Type 3;  1 mg iron/kg DFS. | No color changes in the DFS during transport and storage for 2 years. Iron was found to be stable. No complaints regarding taste, but food turned slightly sour when kept for more than 6 hrs (determined using questionnaires to heads of households and the women in families). | The iron and iodine in the DFS were stable during storage for 2 years - no significant difference (p > .05) between the stability of iodine in DFS and in iodized salt. |
| Rajagopalan & Vinodkumar 2000(29) | India | Double-blind randomized efficacy trial where DFS was distributed to half the adult men and women tea plantation workers and common unfortified salt distributed to the other half. The tea estate is isolated and far from the city, therefore participants could be closely monitored and access to non-study salt was difficult. Duration 12 months. | Type 3;  1 mg iron/kg DFS. | No change in colour, taste, or appearance of the food when cooking with DFS. | Samples were collected from the kitchens of the end user and tested for stability of iron and iodine; iron and iodine were stable in these samples for more than a year. |
|  |  |  |  |  |  |
|  |  |  |  |  |  |
| Zimmermann et al 2002, 2003(49, 50) | Morocco | Double-blind randomized efficacy trial in iodine deficient 6-15 year old children from two neighboring primary schools where intervention group received DFS and control group received iodized salt. Salt was dispensed monthly directly to the head of the household from a central supply at the local health center. Duration 9 months. | Type 4;  1mg iron/kg DFS | After storage for 20 weeks, the DFS and iodized salt were not significantly different in color. Stability was acceptable when the compounds were added to local meals. During the damp winter season, when the moisture content of the local salt is high (~3%), the DFS developed a mild yellow color during storage. | There was no significant difference in the iodine content of the salts during the 20 week period in either the dry or the damp season. In both seasons, the salts each lost about 15% of their iodine content by 20 weeks. |
| Zimmermann et al 2004(51) | Morocco | Double-blind randomized efficacy trial in iodine deficient 6-15 year old children from two neighboring primary schools where intervention group received DFS and control group received iodized salt. Population with a high prevalence of anemia living in rural villages in the Brikcha Rural Commune. Duration 10 months. | Type 5;  2 mg iron/kg DFS | After storage for 6 months, there were no significant differences in color lightness between the DFS and iodized salt. Both salts were universally used, and there were no significant differences between the DFS and the iodized salt in acceptability of salt color, salt taste in foods, and overall acceptability. | There was no significant difference between the DFS and the iodized salt in iodine content during storage; both salts lost about 20% of their iodine content after 6 months. |
| Wegmuller et al 2006(52) | Cȏte d'Ivoire | Double-blind randomized efficacy trial in iron-deficient (with or without anemia) children 5-15 years of age in rural village in Dabou district where intervention group received DFS and control group received iodized salt. All children dewormed at baseline. Duration 6 months. | Type 5;  3 mg iron/kg DFS | No difference in color lightness between DFS and iodized salt. In the triangle testing comparing DFS and iodized salt, there was no detectable difference in color, odor, or taste in either the traditional staples (rice, cassava, yam, plantain) or the sauces (tomato, eggplant, okra, palm nut). | DFS and iodized salt were stored as 10- and 5-kg portions in loosely woven, high-density polyethylene bags typically used to package salt at the production site and as two 300-g portions in transparent low-density polyethylene bags typically used at the retail level and in markets. Iodine loss at 6 mo was ~50% for the iodized salt and ~70% for the DFS when stored in low-density polyethylene bags and close to 100% for both salts when stored in high-density polyethylene bags. |
| Kaur 2000 (53) | India | Efficacy study in 150 young women (healthy, non-pregnant, non-lactating) residing in the University Girls' Hostel of Punjab Agricultural University, Ludhiana, who received DFS or iodized salt. All subjects were dewormed prior to enrolment. No description of randomized design. Duration 6 months. | Not reported | - | Iodine content in DFS decreased upon storage for 6 months in closed containers - the percent decrease was by 41.4% in polyethylene containers, 24.1% in steel containers and 20.7% in air-tight plastic containers respectively. Significant (p<0.01) decrease in iodine content of salt was observed at 4 months of storage in all the storage containers. The iodine content of salt packed in polythene (13.6 ppm) decreased below 15 ppm (minimum recommended level of iodine in salt at retail level) after 6 months of storage, whereas it remained above 15 ppm in the other two storage containers. |
| Working Group on Fortification of Salt with Iron 1982(35) | India | Non-randomized trial in four areas of India (3 rural and 1 urban) where villages assigned to receive DFS or nothing for 12 months. In one area, DFS was sold through village shops; in the other three areas, DFS was distributed to households free of charge. New Delhi participants were not followed up. In Calcultta, results were confounded by deworming and so are not presented. Only change in hemoglobin was presented with no baseline values and therefore, hemoglobin is not presented here. Pre and post intervention anemia prevalence was only presented for the intervention group. Duration 12 months. | Ferric orthophosphate. 1000 mg iron/kg DFS | Based on previous studies, good acceptability and bioavailability of iron. |  |
|  |  |  |  |  |  |
| Nadiger et al 1980(34) | India | Non randomized trial in school-aged children where one school received DFS and one received crushed salt. Group heamoglobin was not comparable at baseline. Duration 12 months. | Not reported | The fortified salt was found to be acceptable in colour and taste when added to diets cooked by traditional methods. Bioavailability of iron did not alter on storage under hot humid conditions. | - |
|  |  |  |  |  |  |

Type 1a DFS refers to DFS which contains microencapsulated potassium iodide and ferrous fumarate; Type 1b contains encapsulated ferrous fumarate; Type 2 contains a refined iodized salt, ferrous sulfate, and a stabilizing compound; Type 3 contains ferrous sulfate with various chelating agents and encapsulated iodine; Type 4 contains encapsulated ferrous sulfate; and Type 5 contains micronized ferric pyrophosphate. DFS, double fortified salt; EFF, encapsulated ferrous fumarate; Hb, hemoglobin; m, months; RCT, randomized controlled trial; SAC, school-age children.

Supplemental Table 2: Summary of double fortified salt study outcomes

| **Author and year** | **Coverage** | **DFS intake (g/(person·d))** | **Awareness creation** | **Safety** | **Population group** | **Baseline mean Hb (g/dL) and anemia %** | **Sample size** | **Endline hemoglobin concentration (mean±SD unless otherwise reported , g/dL); ferritin concentration (mean±SD unless otherwise reported, ug/L); urinary iodine concentration (median [range], ug/L); anemia (%); IDA (%)** | **Global quality rating** |
| --- | --- | --- | --- | --- | --- | --- | --- | --- | --- |
| Asibey-Berko et al 2007(42) | - | 10 | Weekly visits by field workers to encourage compliance with DFS. | No adverse side effects such as nausea, vomiting, or epigastric discomfort were reported by parents. Nor tremors or signs of hyperthyroidism. | children | Hb: 11.0; Anemia: 26.7% | N INT=23; N CTL=59 | *Hb*: INT 11.20±1.20; CTL 10.80±1.50; *Anemia*: INT 8; CTL 35 | Weak |
|  |  |  |  |  | women | Hb: 12.6; Anemia: 16.0% | N INT=65; N CTL=58 | *Hb*: INT 12.40±1.30; CTL 12.3±1.10; *Anemia*: INT 23; CTL 20 |  |
| Nti-Nimako 1998(43) | - | 11.29 | - | - | children and adolescents | Hb: 11.1; Anemia: 51% | N INT=37; N CTL=36 | Hb : INT 11.30; CTL 11.00; Ferritin: INT 67.6; CTL 55.2; Iodine: INT 276; CTL 216; Anemia: INT 19; CTL 36 | Weak |
| Nair et al 2013(32) | - | - | - | - | children | Hb: 11.1 | N Total=3125 | *Hb*: INT 11.6 | Weak |
| Andersson et al 2008(20) | - | 8.3 | - | - | children and adolescents | Hb: 12.5; Anemia: 16.2% | N INT=155; N CTL=151 | *Hb*: INT 13.30±1.20; CTL 13.00±1.40; *Ferritin*: INT 19.7±17.2; CTL 11.6±12.3; *Iodine*: INT 166[17-723]; CTL 355[33-1223]; *Anemia*: INT 7.7; CTL 14.5; *IDA*: INT 6.4; CTL 15.2 | Moderate |
|  | - | 8.3 | - | - | children and adolescents | Hb: 12.5; Anemia: 17.1% | N INT=152; N CTL=151 | *Hb*: INT 13.40±1.10; CTL 13.00±1.40; *Ferritin*: INT 19.3±15.3; CTL 11.6±12.3; *Iodine*: INT 252[14-1156]; CTL 355[33-1223]; *Anemia*: INT 5; CTL 14.5; *IDA*: INT 3.8; CTL 15.2 |  |
| Jayatissa et al 2012(44) | - | - | - | - | children | Hb: 12.3; Anemia: 22.6% | N INT=338; N CTL=336 | *Hb*: INT 12.19±0.92; CTL 12.08±0.87; *Ferritin*: INT 37.14±29,29; CTL 27.18±22.25; *Iodine*: INT 164[4-628]; CTL 134[6-475]; *Anemia*: INT 20.1; CTL 21.2 | Weak |
| Haas et al 2014(45) | Not described, but no other salt was sold in the tea estate, so unlikely that other salt than study salt was used by the participants. | 12.4-15 | - | - | women | Hb: 11.7; Anemia: 53% | N INT=104; N CTL=108 | Hb: INT 11.70±1.20; CTL 11.50±1.10; Ferritin: INT 44.6±30.2; CTL 39.7±34.6; Anemia: INT 54; CTL 68 | Strong |
| Banerjee et al 2018(31) | 42.5% of households in sales villages ever tried DFS, and 14.5% were using it at endline survey | - | Conducted information campaigns, and in some of them, used additional randomized interventions to study how to increase sales (including edutainment movie and providing shopkeepers incentives to sell the DFS). Social marketing experiments induced greater take up in some villages than in some others. In particular, DFS take up (measured as "currently using DFS") was about 5 percentage points higher in villages where showed a high production value "edutainment" movie, and in villages where shopkeepers were given an incentive to market DFS. | - | all | Hb: 12.2; Anemia: 44.7% | N Total=34732 | *Hb*: 𝛃±SE: 0.033±0.029; *Anemia*: 𝛃±SE: -0.006±0.009 | Weak |
|  |  |  |  |  | infants | - | N Total=1242 | *Hb*: 𝛃±SE: -0.032±0.08; *Anemia*: 𝛃±SE: 0.004±0.024 |  |
|  |  |  |  |  | SAC |  | N Total=12775 | Hb: 𝛃±SE: 0.064±0.036; Anemia: 𝛃±SE: -0.02±0.013 |  |
|  |  |  |  |  | adults |  | N Total=15576 | *Hb*: 𝛃±SE: 0.037±0.038; *Anemia*: 𝛃±SE: -0.007±0.01 |  |
|  |  |  |  |  | elderly |  | N Total=6295 | *Hb*: 𝛃±SE: -0.041±0.047; *Anemia*: 𝛃±SE: 0.021±0.013 |  |
|  |  |  |  |  | women |  | N Total=8772 | *Hb*: 𝛃±SE: 0.016±0.038; *Anemia*: 𝛃±SE: -0.001±0.013 |  |
|  | 61% of households were using it at the time of the survey, and 75% of households had been using it (many of the others had just recently run out). | - | - | - | all | - | N Total=21623 | *Hb*: 𝛃±SE: 0.045±0.048; *Anemia*: 𝛃±SE: -0.015±0.015 |  |
|  |  |  |  |  | infants | - | N Total=780 | *Hb*: 𝛃±SE: -0.018±0.11; *Anemia*: 𝛃±SE: 0.015±0.045 |  |
|  |  |  |  |  | SAC | - | N Total=7960 | *Hb*: 𝛃±SE: 0.109±0.058; *Anemia*: 𝛃±SE: -0.032±0.02 |  |
|  |  |  |  |  | adults | - | N Total=9670 | *Hb*: 𝛃±SE: 0.032±0.056; *Anemia*: 𝛃±SE: -0.008±0.016 |  |
|  |  |  |  |  | elderly | - | N Total=3925 | *Hb*: 𝛃±SE: -0.122±0.084; *Anemia*: 𝛃±SE: 0.006±0.023 |  |
|  |  |  |  |  | women | - | N Total=5455 | *Hb*: 𝛃±SE: 0.059±0.062; *Anemia*: 𝛃±SE: -0.009±0.02 |  |
| Sivakumar et al 2001(25) | 100% | 7-9 | - | Sodium hexa meta phosphate used at 1% levels as a stabilizer may influence the calcium and phosphorous homeostasis. However, no complaints were registered and no clinical signs of adverse effects were observed among consumers during period of supplementation. | All | - | N INT=689; N CTL=702 | *Iodine*: INT 155; CTL 97 | Weak |
|  |  |  |  |  | infants | Hb: 10.25 | N INT=360; N CTL=369 | *Hb*: INT 11.20±1.30; CTL 11.30±1.69 |  |
|  |  |  |  |  | SAC | Hb: 10.87 | N INT=232; N CTL=261 | *Hb*: INT 11.60±1.56; CTL 12.00±1.67 |  |
|  |  |  |  |  | adolescents | Hb: 11.77 | N INT=37; N CTL=32 | *Hb*: INT 12.52±2.00; CTL 12.15±2.02 |  |
|  |  |  |  |  | adolescent girls | Hb: 11.22 | N INT=19; N CTL=24 | *Hb*: INT 11.40±2.20; CTL 12.00±1.93 |  |
|  |  |  |  |  | adolescent boys | Hb: 12.31 | N INT=18; N CTL=8 | *Hb*: INT 13.70±1.79; CTL 12.6±2.29 |  |
|  |  |  |  |  | pregnant women | Hb: 9.36 | N INT=25; N CTL=14 | *Hb*: INT 10.40±1.62; CTL 9.90±1.73 |  |
|  |  |  |  |  | lactating women | Hb: 10.52 | N INT=35; N CTL=26 | *Hb*: INT 11.40±1.16; CTL 10.9±2.15 |  |
| Kramer et al 2018(30) | Not reported, but likely close to 100% because few shortages and monitoring visits indicate that schools were using DFS in midday meal. | - | - | - | SAC | Hb: 11.5; Anemia: 45.5% | N INT=726; N CTL=680 | *Hb*: 𝛃±SE: 0.14±0.08; *Anemia*: 𝛃±SE: 0.09±0.03 | Moderate |
| Reddy & Nair 2014(27) | - | - | - | - | children and adolescents | Hb: 9.17; Anemia: 99% | N INT=442; N CTL=505 | *Hb*: INT 9.09±0.87; CTL 9.08±0.91; *Iodine*: INT 183.37; CTL 244.57; *Anemia*: INT 83.4; CTL 84.8 | Weak |
| Sivakumar et al 2001(25) | 100% | 7-9 | - | Sodium hexa meta phosphate used at 1% levels as a stabilizer may influence the calcium and phosphorous homeostasis. However, no complaints were registered and no clinical signs of adverse effects were observed among consumers during period of supplementation. The urinary levels of calcium and phosphorus in DFS-fed children were also not different from those of iodized salt children indicating that consumption of DFS containing a polyphosphate stabilizer for 2 years did not alter calcium and phosphorus homeostasis. | SAC | Hb:12.02; Anemia: 39.1% | N INT=448; N CTL=352 | *Hb*: INT 11.80±1.98; CTL 10.7±2.07; *Iodine*: INT 108[33-249]; CTL 452[116-1081] | Weak |
| Bathla & Grover 2017(46) | - | - | Experimental group was provided with nutrition education every 15 days for 3 months in the form of printed materials on food groups, balanced diet, function of different nutrients and their requirements, nutritional disorders, their control and prevention, use and storage of salt, and healthy cooking practices. | - | adolescent girls | Hb: 9.9; Anemia: 100% | N INT=30; N CTL=30 | *Hb*: INT 10.48±0.80; CTL 10.33±0.64; *Iodine*: INT 108; CTL 103; *Anemia*: INT 90; CTL 100 | Weak |
| Reddy & Nair 2016(47) | - | 10 | - | - | pregnant women | Hb: 9.40; Anemia: 87.4% | N INT=67; N CTL=54 | Hb: INT 9.86±1.00; CTL 9.15±1.00; *Iodine*: INT 299[121-493]; CTL 289[107-783]; *Anemia*: INT 88.1; CTL 96.3 | Weak |
| Nair et al 2014(33) | - | - | - | - | pregnant women | Hb: 4.8; Anemia: 100% |  | *Hb*: INT 8.3; *Iodine*: INT 106 | Weak |
| Vinodkumar et al 2007(48) | - | 10 | - | - | children, adolescents, and adults | Hb: 10.3 | N INT=393; N CTL=436 | *Hb*: INT 12.32±1.93; CTL 11.06±2.59; *Iodine*: INT 205[70-600]; CTL 220[60-600] | Weak |
| Rajagopalan et al 2000(29) | 100%. Remote area meant that it was difficult to obtain salt other than the study salt. Periodic surprise visits indicated that participants were using only study salt. | 10 | The plantation workers were used to crystal salt, not powdered salt. Talks and cooking demonstrations on how to use the powdered salt were given. Participants were instructed that the salt had nutritional benefits. | Not described | adults | Hb: 9.08 | N INT=385; N CTL=408 | *Hb*: INT 10.19±1.42; CTL 9.99±1.37 | Weak |
|  |  |  |  |  | women | Hb: 8.48 | N INT=230; N CTL=250 | *Hb*: INT 10.03±1.34; CTL 9.75±1.32 |  |
|  |  |  |  |  | men | Hb: 9.57 | N INT=155; N CTL=158 | *Hb*: INT 10.42±1.53; CTL 10.30±1.46 |  |
| Zimmermann et al 2002, 2003(49, 50) | - | 7-12 | At baseline, the study was carefully explained to the participating families, and it was emphasized that the new salt should be used for all cooking and food preparation, as well as at the table. This message was reinforced at each of the monthly salt distributions. | - | children and adolescents | Hb: 11.2 | N INT=183; N CTL=184 | *Hb*: INT 12.70±1.20; CTL 11.60±1.20; *Ferritin*: INT 40.0±25.0; CTL 17.0±12.0; *Iodine*: INT 189[23-406]; CTL 182[14-474]; *IDA*: INT 8; CTL 30 | Strong |
| Zimmermann et al 2004(51) | - | 7-12 | At baseline, the study was carefully explained to the participating families, and it was emphasized that the new salt should be used for all cooking and food preparation, as well as at the table. This message was reinforced at each of the monthly salt distributions. | - | children and adolescents | Hb:11.5; Anemia: 79.0% | N INT=75; N CTL=83 | *Hb*: INT 12.80±1.10; CTL 11.50±0.80; *Ferritin,* Geometric mean±SD: INT 33.1±43.3; CTL 15.0±13.1; *Iodine*: INT 97[17-1356]; CTL 104[22-1784]; *Anemia*: INT 12; CTL 58; *IDA*: INT 5; CTL 29 | Strong |
| Wegmuller et al 2006(52) | Salt was used to prepare sauces and the main staples in 92–100% of the households | 4-6.1 | - | - | children and adolescents | Hb: 11.6; Anemia: 52% | N INT=60; N CTL=63 | *Hb*: INT 11.70±1.20; CTL 11.30±1.10; *Ferritin*: INT 63.0±57.2; CTL 61.0±151.9; *Anemia*: INT 47; CTL 62; *IDA*: INT 23; CTL 28 | Moderate |
| Kaur 2000 (53) | - | 8-10 | - | - | women | Hb: 10.9; Anemia: 74.7% | N INT=100; N CTL=50 | *Hb*: INT 12.23±1.20; CTL 11.41±1.56; *Ferritin*: INT 23.1±41.4; CTL 16.3±25.9; *Anemia*: INT 41; CTL 64 | Weak |
| Working Group on Fortification of Salt with Iron 1982(35) | - | - | - | - | Hyderabad - Infants and children | Anemia: 66.3% | N INT=181 | *Anemia*: INT 28.2 | Weak |
|  |  |  |  |  | Madras - Infants and children | Anemia: 19.1% | N INT=110 | *Anemia*: INT 9.1 |  |
|  |  |  |  |  | Hyderabad - Male children | Anemia: 52.9% | N INT=189 | *Anemia*: INT 27 |  |
|  |  |  |  |  | Madras - Male children | Anemia: 15.9% | N INT=182 | *Anemia*: INT 10.4 |  |
|  |  |  |  |  | Hyderabad - Female children | Anemia: 63.8% | N INT=185 | *Anemia*: INT 33.5 |  |
|  |  |  |  |  | Madras - Female children | Anemia: 12.4% | N INT=202 | *Anemia*: INT 6.9 |  |
|  |  |  |  |  | Hyderabad - Male adolescents | Anemia: 42.5% | N INT=84 | *Anemia*: INT 20 |  |
|  |  |  |  |  | Madras - Male adolescents | Anemia: 6% | N INT=67 | *Anemia*: INT 4.5 |  |
|  |  |  |  |  | Hyderabad - Female adolescents | Anemia: 73.9% | N INT=92 | *Anemia*: INT 51.1 |  |
|  |  |  |  |  | Madras - Female adolescents | Anemia: 22.3% | N INT=139 | *Anemia*: INT 8.6 |  |
|  |  |  |  |  | Hyderabad - Adult men | Anemia: 28.6% | N INT=178 | *Anemia*: INT 22.5 |  |
|  |  |  |  |  | Madras - Adult men | Anemia: 6.5% | N INT=77 | *Anemia*: INT 6.5 |  |
|  |  |  |  |  | Hyderabad - Adult women | Anemia: 66.8% | N INT=232 | *Anemia*: INT 43.6 |  |
|  |  |  |  |  | Madras - Adult women | Anemia: 33.8% | N INT=196 | *Anemia*: INT 15.8 |  |
|  |  |  |  |  | Hyderabad - ≥ 45 y | Anemia: 48% | N INT=260 | *Anemia*: INT 29.3 |  |
|  |  |  |  |  | Madars –  ≥ 45 y | Anemia: 15.8% | N INT=114 | *Anemia*: INT 9.7 |  |
| Nadiger et al 1980(34) | School children were not allowed to leave the school premises and all food eaten by the children came from the community kitchen run by the school, so little opportunity to consume food from outside. | 15 | - | - | SAC boys | Hb: 12.2; Anemia: 50.5% | N INT=222; N CTL=92 | *Hb*: INT 13.4±1.49; CTL 12.0±1.44; *Anemia*: INT 19.4; CTL 51.9 | Weak |
|  |  |  |  |  | SAC girls | Hb: 13.4; Anemia: 21.1% | N INT=161; N CTL=71 | *Hb*: INT 14.8±1.40; CTL 13.1±1.60; *Anemia*: INT 3.0; CTL 22.5 | Weak |

DFS, double fortified salt; Hb, hemoglobin, RCT, randomized controlled trial; SAC, school-age children.

Supplemental Table 3: List of assessment methods used in studies that were included in the review of the efficacy and effectiveness of double fortified salt studies

| **Nutritional marker** | **N** |
| --- | --- |
| ***Hemoglobin*** |  |
| Cyanmethemoglobin method | 11 |
| Coulter Counter | 5 |
| HemoCue | 4 |
| Sahli’s method (54) | 1 |
| Not reported | 1 |
| ***Ferritin*** |  |
| Enzyme-linked immunosorbent assays (ELISA) | 5 |
| Automated chemiluminescent immunoassay system | 2 |
| Melotest Ferritin kit | 1 |
| ***Urinary iodine*** |  |
| Lauber 1975 kinetic method (55) | 2 |
| Pino modification of the Sandell-Kolthoff reaction (56) | 3 |
| Ohashi and Karmarkar et al. modification of Sandell-Kolthoff method (57) | 3 |
| Dunn et al. method (58) | 1 |
| Not reported | 1 |

Supplemental Figure 1: Funnel plot for effect of double fortified salt on hemoglobin concentration (standardized mean difference)


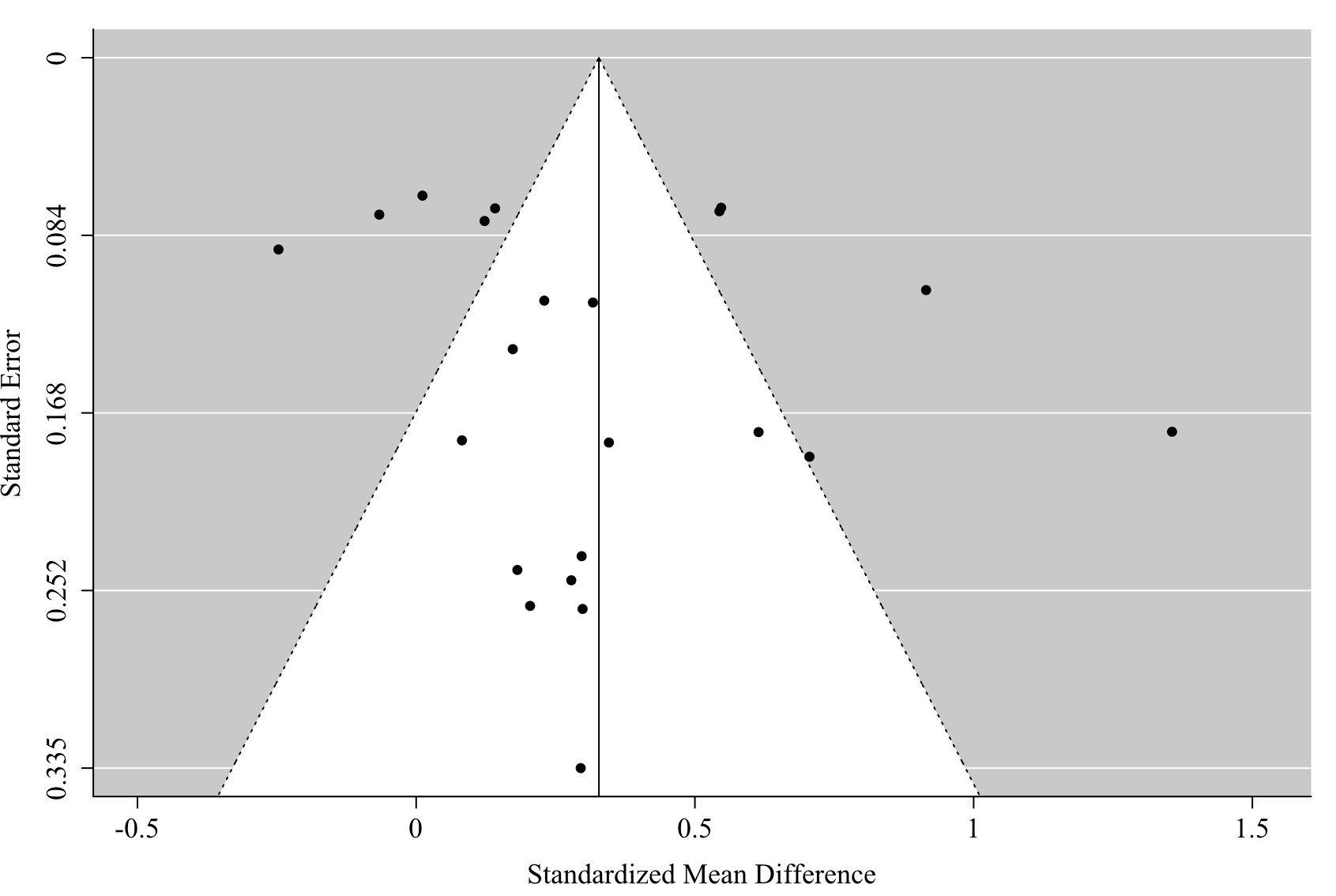


Supplemental Figure 2: Funnel plot for effect of double fortified salt on hemoglobin concentration (mean difference)


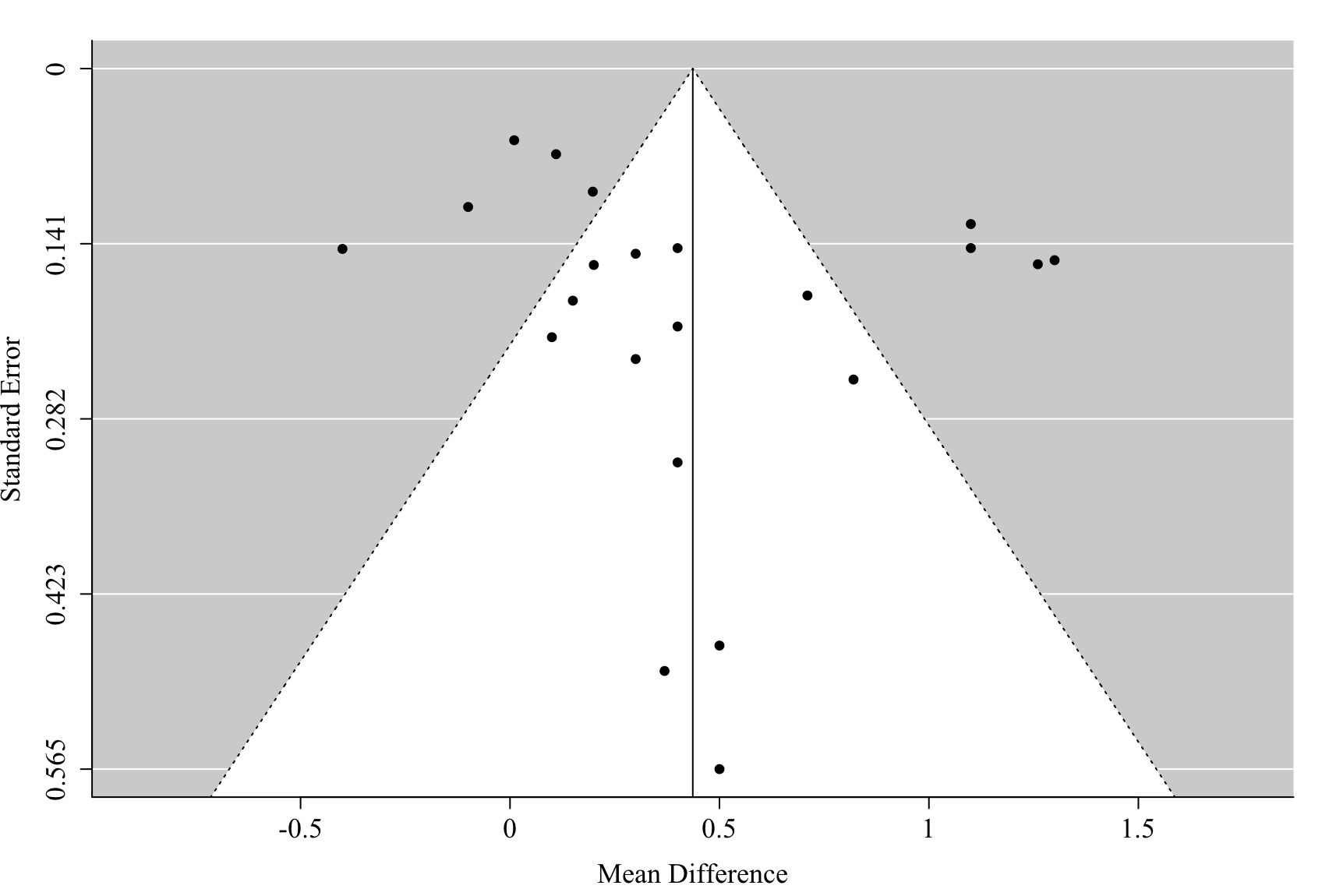


Supplemental Figure 3: Funnel plot for effect of double fortified salt on ferritin concentration (standardized mean difference)


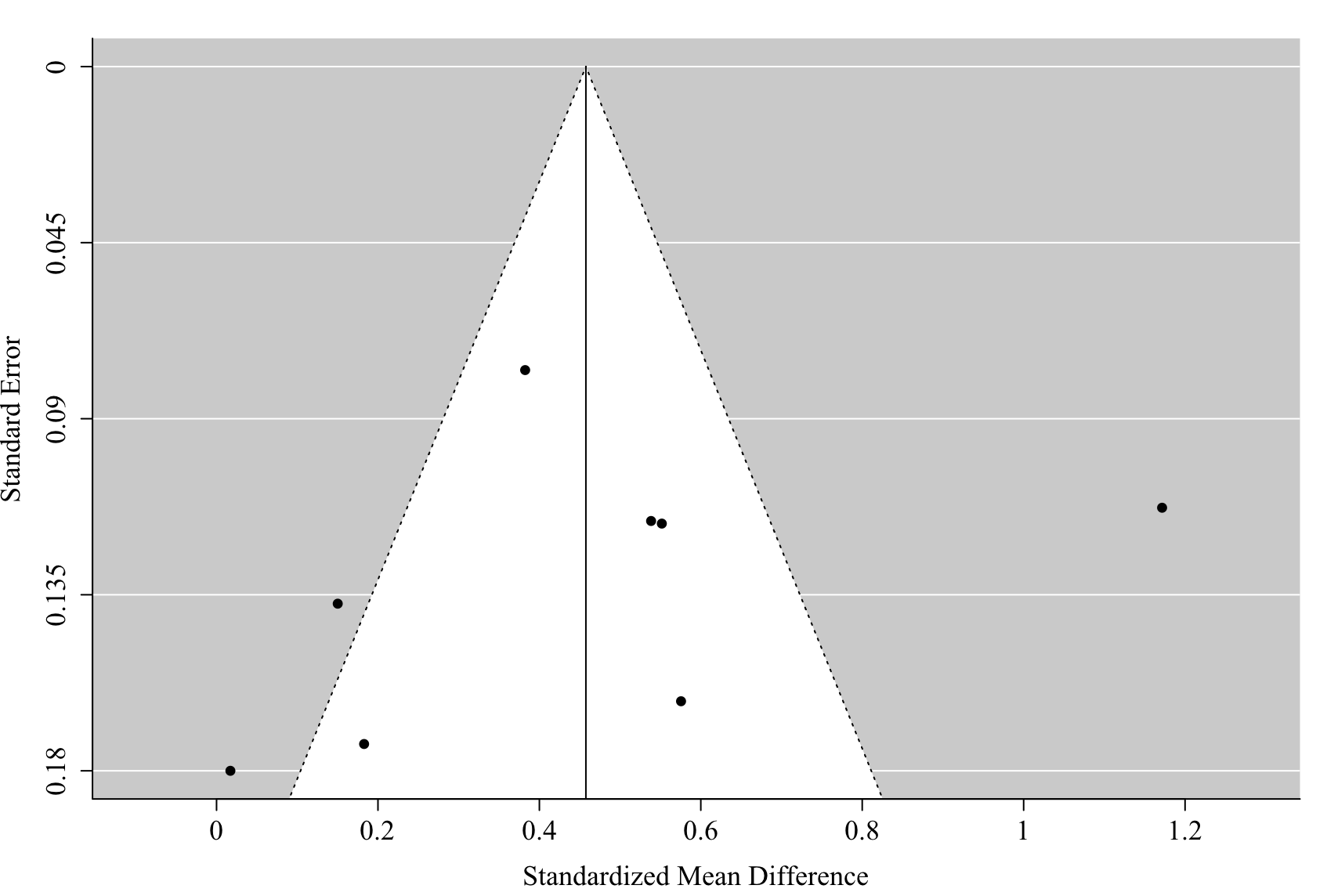


Supplemental Figure 4: Funnel plot for effect of double fortified salt on urinary iodine concentration (standardized mean difference)


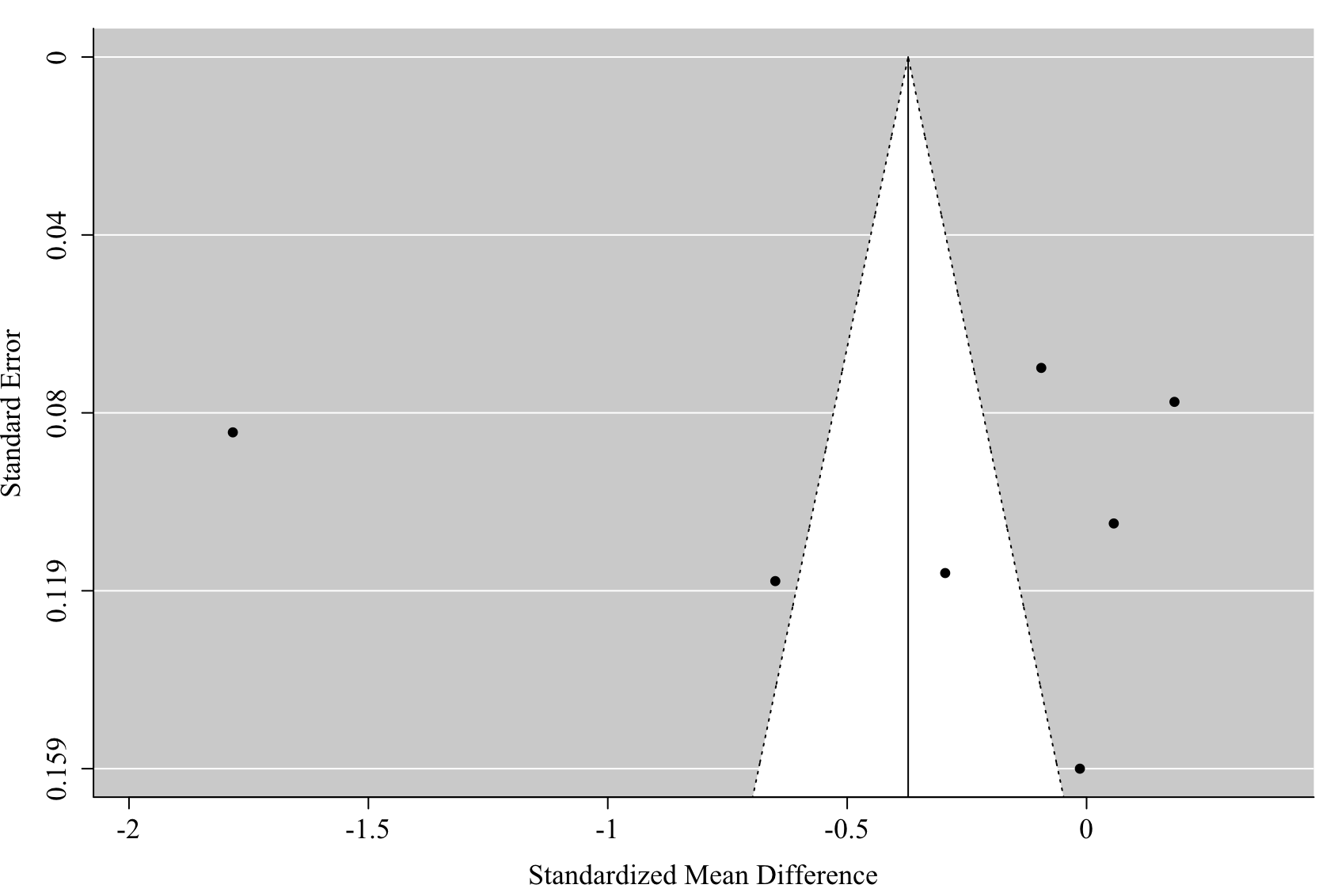


Supplemental Figure 5: Funnel plot for effect of double fortified salt on anemia (risk ratio)


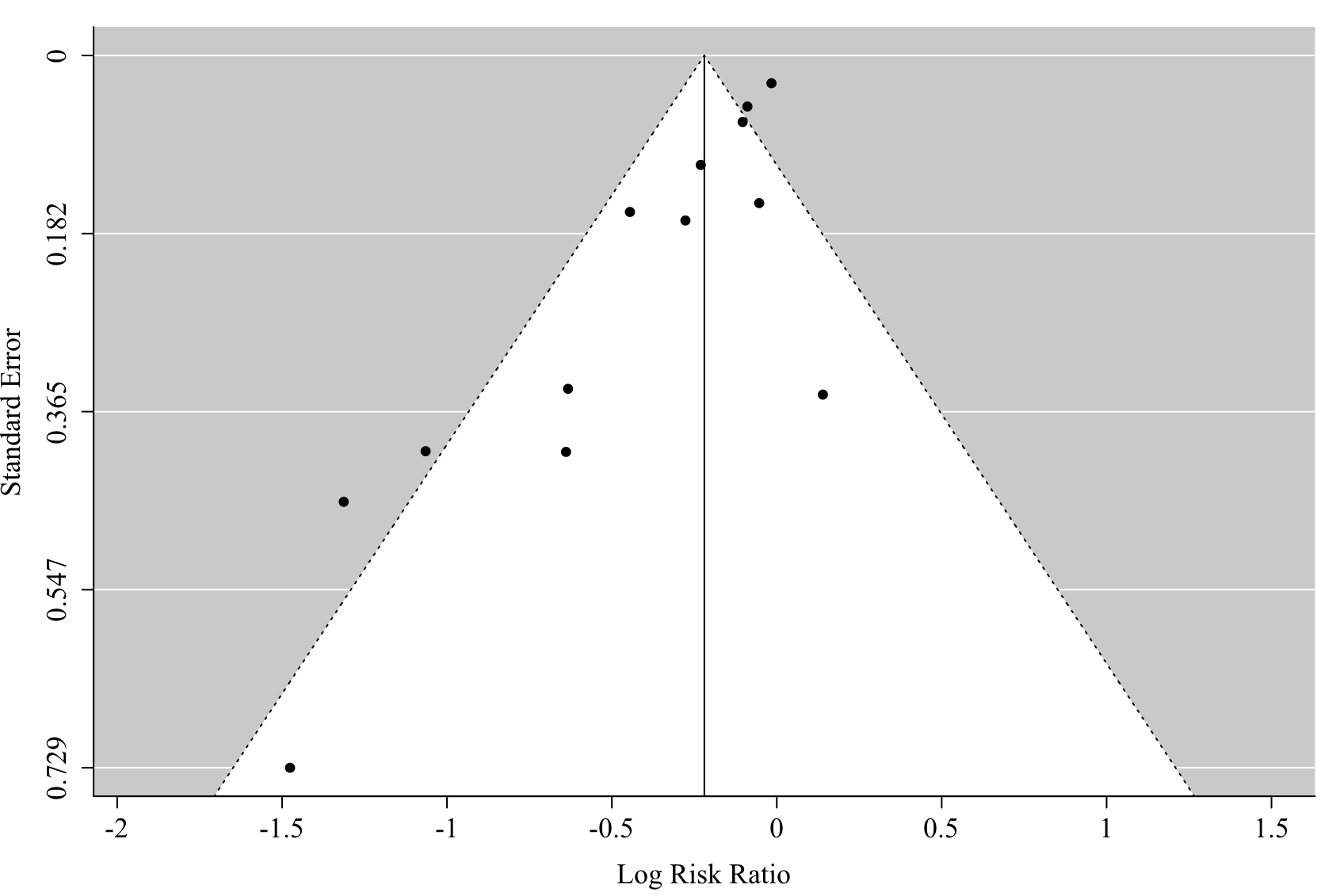


Supplemental Figure 6: Funnel plot for effect of double fortified salt on iron deficiency anemia (risk ratio)


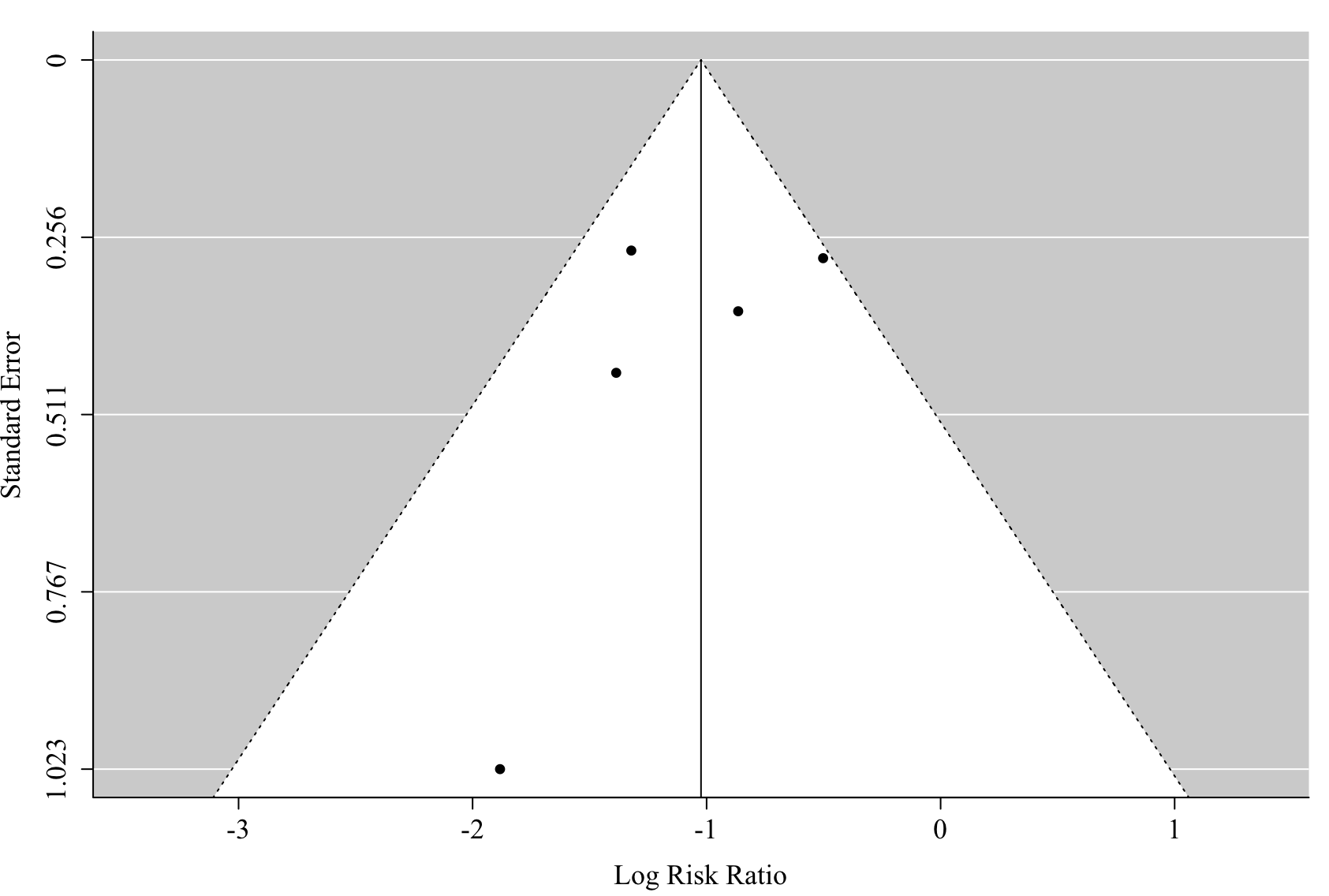


Supplemental Figure 7: Forest plot for effect of double fortified salt on hemoglobin concentration (standardized mean difference)


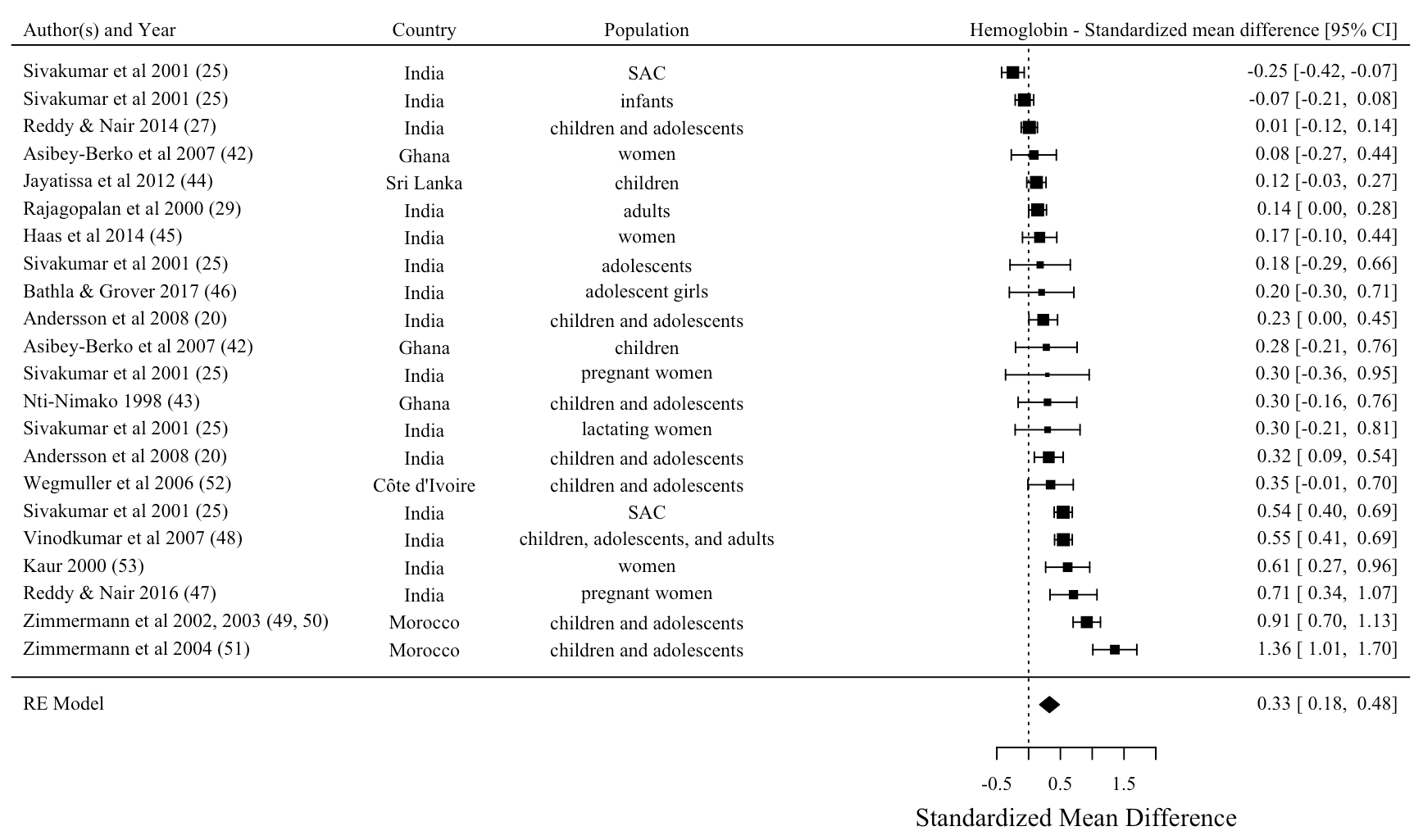


I^2^=89.46%, Q(df=21)= 88.7989, P < 0.0001

Supplemental Figure 8: Forest plot for effect of double fortified salt on urinary iodine concentration (standardized mean difference)


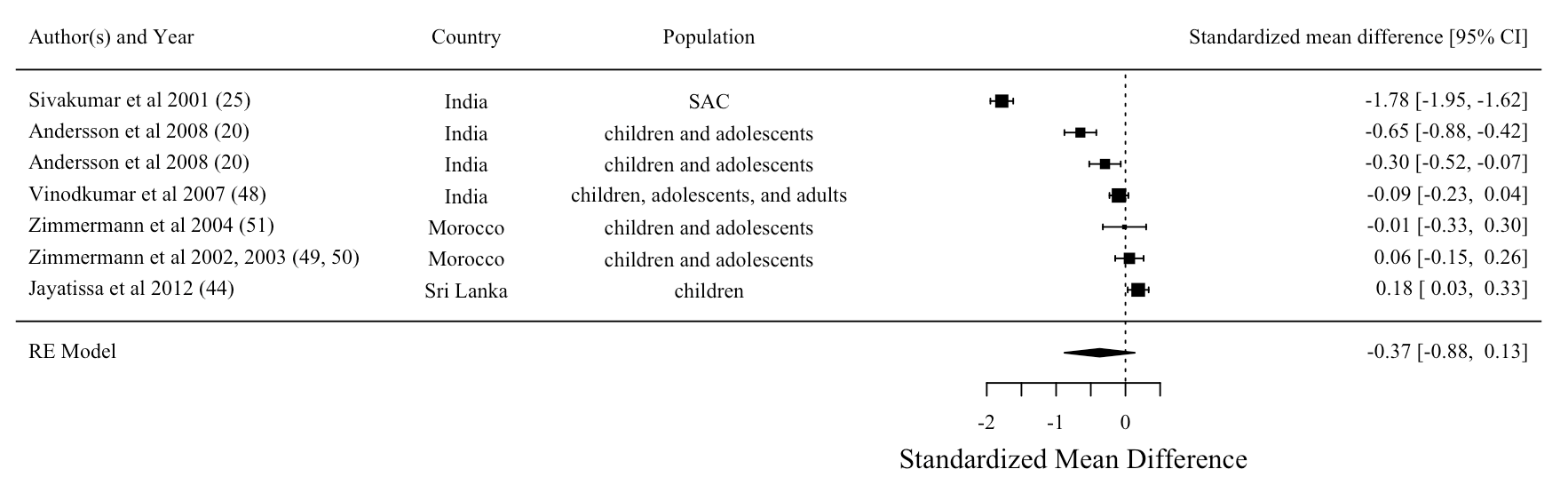
I^2^=98.03%, Q(df=6)= 377.5454, P <0 .0001
